# Serum miRNA profiles are altered in patients with primary sclerosing cholangitis receiving high-dose ursodeoxycholic acid

**DOI:** 10.1016/j.jhepr.2023.100729

**Published:** 2023-03-23

**Authors:** Jessica T. Hochberg, Aalam Sohal, Priya Handa, Bryan D. Maliken, Take-Kyun Kim, Kai Wang, Eric Gochanour, Yu Li, J. Bart Rose, James E. Nelson, Keith D. Lindor, Nicholas F. LaRusso, Kris V. Kowdley

**Affiliations:** 1 Liver Institute Northwest, Seattle, WA, USA; 2 Seattle Children’s Hospital/University of Washington, Seattle, WA, USA; 3 Miami Transplant Institute at University of Miami, Miami, FL, USA; 4 Benaroya Research Institute, Seattle, WA, USA; 5 Institute for Systems Biology, Seattle, WA, USA; 6 Division of Gastroenterology and Hepatology, Mayo Clinic Rochester, MN, USA

**Keywords:** Serum, miRNA, Biomarker, PSC, UDCA, High-dose

## Abstract

**Background & Aims:**

Primary sclerosing cholangitis (PSC) is a chronic, progressive cholestatic liver disease that can lead to end-stage liver disease and cholangiocarcinoma. High-dose ursodeoxycholic acid (hd-UDCA, 28–30 mg/kg/day) was evaluated in a previous multicentre, randomised placebo-controlled trial; however, the study was discontinued early because of increased liver-related serious adverse events (SAEs), despite improvement in serum liver biochemical tests. We investigated longitudinal changes in serum miRNA and cytokine profiles over time among patients treated with either hd-UDCA or placebo in this trial as potential biomarkers for PSC and response to hd-UDCA, as well as to understand the toxicity associated with hd-UDCA treatment.

**Methods:**

Thirty-eight patients with PSC were enrolled in a multicentred, randomised, double-blinded trial of hd-UDCA *vs.* placebo.

**Results:**

Significant alterations in serum miRNA profiles were found over time in both patients treated with hd-UDCA or placebo. Additionally, there were striking differences between miRNA profiles in patients treated with hd-UDCA compared with placebo. In patients treated with placebo, the changes in concentration of serum miRNAs miR-26a, miR-199b-5p, miR-373, and miR-663 suggest alterations of inflammatory and cell proliferative processes consistent with disease progression**.** However, patients treated with hd-UDCA exhibited a more pronounced differential expression of serum miRNAs, suggesting that hd-UDCA induces significant cellular miRNA changes and tissue injury. Pathway enrichment analysis for UDCA-associated miRNAs suggested unique dysregulation of cell cycle and inflammatory response pathways.

**Conclusions:**

Patients with PSC have distinct miRNAs in the serum and bile, although the implications of these unique patterns have not been studied longitudinally or in relation to adverse events related to hd-UDCA. Our study demonstrates marked changes in miRNA serum profiles with hd-UDCA treatment and suggests mechanisms for the increased liver toxicity with therapy.

**Impact and implications:**

Using serum samples from patients with PSC enrolled in a clinical trial comparing hd-UDCA with placebo, our study found distinct miRNA changes in patients with PSC who are treated with hd-UDCA over a period of time. Our study also noted distinct miRNA patterns in patients who developed SAEs during the study period.

## Highlights

• Longitudinal changes occurred in miRNA profiles of patients treated with hd-UDCA.
• The concentration of miR-92b, an oncogene, increased in patients treated with hd-UDCA.
• The concentration of miR-122, miR-885-5p, miR-375, and miR-99a decreased in patients treated with hd-UDCA.
• Patients who progressed to liver failure resulting in liver-related death or liver transplantation demonstrated a unique miRNA pattern with 18 DE-miRNAs.

## Graphical abstract

**Figure:**
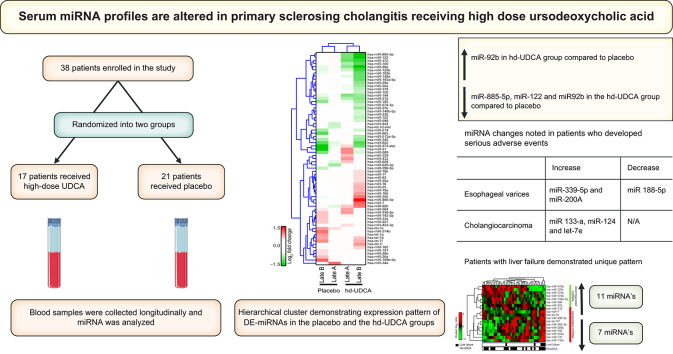

## Introduction

Primary sclerosing cholangitis (PSC) is a progressive cholestatic liver disease of unknown etiopathogenesis, which can lead to end-stage liver disease, portal hypertension, and cholangiocarcinoma (CC), with a median survival of 15–20 years following diagnosis.^1^ There are no approved therapies for PSC, with liver transplant being the only definitive therapy currently.

Ursodeoxycholic acid (UDCA), a secondary bile acid, has been used to treat cholestatic liver diseases. UDCA (13–15 mg/kg/day) is approved by the FDA for the treatment of primary biliary cholangitis, but not primary sclerosing cholangitis. The efficacy of high-dose UDCA (hd-UDCA, 28–30 mg/kg/day) as treatment for PSC was evaluated in a multicentre randomised placebo-controlled trial.^2^ Although patients treated with hd-UDCA demonstrated improvement in serum liver biochemical tests, the trial was prematurely terminated because of an unexpected higher rate of liver-related serious adverse events (SAEs), including liver-related death, liver failure, and liver transplantation, among patients treated with hd-UDCA. The possible pathophysiological mechanisms for increased liver-related SAEs in patients treated with hd-UDCA have not been elucidated.

miRNAs are small non-coding RNAs that negatively regulate genes by promoting mRNA degradation and/or blocking translational initiation. Recent studies demonstrated the involvement of miRNAs in key liver functions, including the homeostasis of glucose,^3^ cholesterol,^4^ and iron.^5^ Increasingly, miRNAs have been associated with numerous diseases, such as metabolic disorders, cancers, and various liver diseases, including cholangitis,^6^ non-alcoholic fatty liver disease (NAFLD),^7^ HCV,^8^ HBV,^9^ and hepatocellular carcinoma (HCC).^10^ Cellular miRNAs are also released into the circulation, and may represent a new type of biomarker for PSC and other cholestatic liver diseases because they are: (i) protected from the high activity of RNase activities present in the extracellular environment by forming protein complexes or encapsulated in lipid vesicles;^11^ (ii) stable for multiple freeze thaw cycles;^12^ and (iii) readily measurable with common laboratory technologies, including next-generation sequencing (NGS) or quantitative reverse transcription (qRT)-PCR.^13^ Most recently, extracellular vesicles containing miRNA were identified with the hopes of opening new opportunities for non-invasive diagnostic tools for patients with PSC.^14^ Therefore, characterising miRNA profiles may provide novel insights into pathophysiology and mechanisms for liver injury and disease.

The goal of this study was to examine whether there were differences over time in serum miRNA profiles between patients treated with either placebo or hd-UDCA. Our hypothesis was that hd-UDCA therapy in PSC is associated with distinct changes in miRNA profiles compared with placebo.

## Materials and methods

### Patients

Patients were enrolled in a multicentered, randomised, double-blinded trial of hd-UDCA (28–30 mg/kg/day) versus placebo; details of this study have been previously published.^2^ Institutional review board at each site approved the study. Thirty-eight subjects from the original cohort (placebo = 17, hd-UDCA = 21) with available serum samples were included in the present study. Blood samples from each patient from both baseline entry point and a second time point were included. The current study was approved by the institutional review board at Benaroya Research Institute, Seattle, WA and Mayo Clinic, Rochester, MN.

### Serum samples

Blood samples were collected and processed as described previously.^15^ Serum samples were stored in a –80 °C freezer before RNA extraction. We then examined the levels of 375 human miRNAs and 27 cytokines in serum samples from all 38 patients. We identified serum miRNA signatures associated with PSC progression, hd-UDCA treatment, and SAEs, including esophageal varices (EVs), CC, and liver failure.

### RNA extraction

The protocol for RNA extraction from 200-μL serum was described previously.^15^ Synthetic cel-miR-54 (20 *f*mol) (IDT DNA Technologies, IA, USA) was spiked into each serum sample during the RNA extraction process for normalisation. Total RNA extracted from 200-μL serum was eluted from the RNA isolation column in 50-μL ddH_2_O.

### TaqMan miRNA real-time qPCR

TaqMan miRNA real-time qPCR was performed to quantify the cel-miR-54 spike-in control. Briefly, 5 μL of total RNA was reversely transcribed in a 15-μL reaction volume for each sample using the TaqMan miRNA reverse-transcription kit (Thermo Fisher Scientific, San Francisco, CA, USA) according to the manufacturer’s protocol. TaqMan cel-miR-54 probe and TaqMan Universal Master Mix No AmpErase® UNG were purchased from Thermo Fisher Scientific (Thermo Fisher Scientific). Real-time qPCR was performed on an ABI-7500 real-time PCR instrument (Life Technologies) according to the manufacturer’s protocol.

### miRNA profiling

miRNA profiling was performed using the miRCURY LNA™ miRNA real-time PCR Human Panel I (Exiqon, Copenhagen, Denmark). The panel was designed based on the miRBase release version 14 and contained probes for 375 human miRNAs. For miRNA profiling, 15 μL of total RNA was reversely transcribed in a 40-μL reaction volume for each sample. The miRCURY Locked Nucleic Acid (LNA) universal cDNA synthesis kit (Exiqon) was used to make cDNA for miRNA profiling according to the manufacturer’s protocol. Real-time qPCR was performed using the SYBR Green Master Mix (Exiqon) on an ABI-7900HT real-time PCR instrument (Life Technologies) according to the manufacturer’s protocol.

### Cytokine analysis

Twenty-seven cytokines levels were measured using Bio-Plex® Precision Pro™ Human Cytokine Assays (Bio-Rad Laboratories, Hercules, CA, USA) on a Luminex 100 instrument (Luminex, Austin, TX, USA), according to the manufacturers’ protocols. Twenty-seven cytokines were analysed: IL-1β, IL-1ra, IL-6, IL-7, IL-8, IL-13, IL-15, IL-17, IL-18, fibroblast growth factor (FGF)-basic, eotaxin, granulocyte colony-stimulating factor (G-CSF), GRO-α, hepatocyte growth factor (HGF), interferon (IFN)-γ, interferon gamma-induced protein 10 (IP-10), leukemia inhibitory factor (LIF), macrophage migration inhibitory factor (MIF), monocyte chemoattractant protein 1 (MCP-1), macrophage inflammatory protein (MIP)-1α, MIP-1β, platelet-derived growth factor (PDGF)-BB, regulated upon activation, normal T cell expressed and presumably secreted (RANTES), stem cell factor (SCF), tumour necrosis factor (TNF)-α, vascular endothelial growth factor (VEGF), and tumour necrosis factor-related apoptosis-inducing ligand (TRAIL).

### Data analysis

A data matrix comprising cycle threshold (Ct) values for miRNAs and 104 samples was prepared. Average values for miRNAs with Ct <35 were computed at each sample. Mean normalisation was then performed by subtracting the average value from individual miRNA Ct values at each sample. miRNAs with more than 20% missing values across the samples were removed. The remaining missing values were imputed with the impute R/Bioconductor package using the kNN imputation algorithm.^16^ Data quality assessment was then performed with either a scatter plot or Pearson correlation coefficient between samples.

miRNAs differentially expressed between the two groups were identified by LIMMA R/Bioconductor package^17^ with the criteria of *p* <0.05 and log2-fold-change >0.585 for upregulation or <–0.585 for downregulation. Biological process/pathway enrichment analysis was performed with miEAA software.^18^ Changes in serum cytokine concentration between the two groups were assessed using the Mann-Whitney *U* test. A *p* <0.05 was considered statistically significant. k-TOP scoring pairs classifier was performed with switchbox R/Bioconductor package^19^ to identify a set of paired miRNA classifiers.

## Results

### Patient characteristics

There were no significant differences in age, sex, and serum levels of aspartate transaminase (AST), alkaline phosphatase (ALP), and total bilirubin at entry between patients in the hd-UDCA group and the placebo group (Table 1). Patients in the hd-UDCA group showed decreased levels of AST and ALP at post-entry points compared with both their entry time point and matched time points of the placebo group. The placebo group showed no difference in AST, ALP, and total bilirubin when comparing entry to all later time points (Table 2). However, the hd-UDCA group exhibited more SAEs, such as liver failure resulting in liver transplantation (0% *vs.* 9.1%) and liver-related death (0% *vs.* 18.2%), compared with the placebo group (Table 3).

**Table 1:** **Characteristics of patients included in the study**.

Patient characteristics	Placebo (n = 17)	hd-UDCA (n = 21)	*p* value
Age (years)	42.0 (33.5–48.5)	44.0 (37–49)	0.433
Sex (male, %)	68.8	54.5	0.506
Blood chemistry∗			
Aspartate aminotransferase	2.7 (1.6–3.7)	2.6 (1.7–4.0)	0.830
Alkaline phosphatase	3.2 (2.3–4.9)	3.8 (2.7–4.8)	0.702
Total bilirubin	1.0 (0.6–1.2)	0.8 (0.7–1.0)	0.559
Albumin	1.2 (1.1–1.3)	1.2 (1.0–1.2)	0.739

Data are presented as median (36) unless otherwise indicated.

∗ All laboratory values are reported as multiples of upper limits of normal.

**Table 2:** **Serum levels of aspartate transaminase, alanine transaminase, and total bilirubin between two groups**.∗

Study time point	n		Aspartate transaminase			Alanine transaminase			Total bilirubin		
	Placebo	hd-UDCA	Placebo	hd-UDCA	*p* value	Placebo	hd-UDCA	*p* value	Placebo	hd-UDCA	*p* value
Baseline (at entry)	22	16	2.7 (1.6–3.7)	2.6 (1.7–4.0)	0.830	3.2 (2.3–4.9)	3.8 (2.7–4.8)	0.702	1.0 (0.6–1.2)	0.8 (0.7–1.0)	0.559
12 months	21	16	2.7 (1.6–3.3)	1.1 (0.8–2.2)	**0.014**	1.9 (1.5–2.5)	1.9 (1.5–2.5)	**0.003**	1.0 (0.7–1.4)	0.7 (0.6–1.0)	0.115
24 months	22	15	2.4 (1.5–3.3)	1.4 (1.1–2.0)	0.055	1.9 (1.2–2.6)	1.9 (1.2–2.6)	**0.027**	0.9 (0.6–1.6)	0.9 (0.6–1.5)	0.710
36 months	17	15	2.5 (1.6–4.0)	1.3 (0.7–1.8)	**0.007**	1.6 (1–2.5)	1.6 (1.0–2.5)	**0.001**	0.9 (0.6–2.0)	1.0 (0.7–1.4)	0.584

Data are presented as the median (36) of upper limits of normal laboratory value. Bold suggests statistical significance.

∗ Mann-Whitney *U* test was used to compare the groups.

**Table 3:** **Number of patients in the hd-UDCA and placebo groups who progressed to the primary endpoint**.

Primary endpoint	Placebo (n = 17), n (%)	hd-UDCA (n = 21), n (%)
End cirrhosis	1 (4.5)	1 (6.3)
End esophageal varices	5 (22.7)	6 (37.5)
Cholangiocarcinoma	2 (9.1)	2 (12.5)
Transplant	0	2 (12.5)
Death	0	4 (25)

### The placebo group and the hd-UDCA group exhibited distinct time-associated serum miRNA patterns

**Fig. 1:**
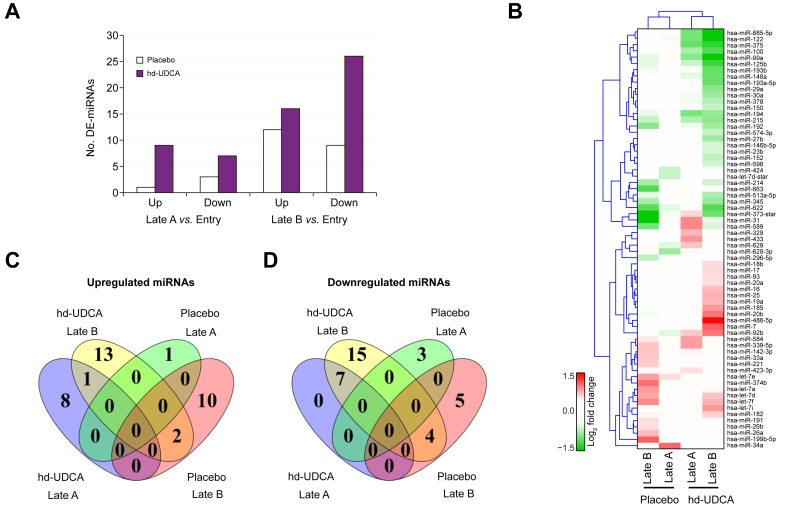
Patients treated with hd-UDCA *vs.* placebo exhibit distinct serum cell-free miRNA patterns. The miRNA signatures with significant expression changes were selected using a cut-off of a fold change >1.5 and *p* <0.05. The changes in miRNA in patients receiving placebo or hd-UDCA in post-entry time points are relative to the corresponding entry time point. (A) Numbers of miRNA with significant changes in concentration at different post-entry time points. (B) Unsupervised hierarchical cluster demonstrating the expression pattern of DE-miRNAs in the placebo and hd-UDCA groups at different post-entry time points. Red represents increased concentrations; green represents decreased concentrations. A total of 67 DE-miRNAs passed the cut-off and were included in the analysis. (C). Relationships of DE-miRNAs at increased concentrations in hd-UDCA or placebo compared with baseline sample at entry. (D) Relationships of DE-miRNAs at decreased concentration in either hd-UDCA or placebo groups compared with sample at entry. DE, differentially expressed; hd-UDCA, high-dose ursodeoxycholic acid.

We compared the post-entry serum miRNA profiles of patients receiving placebo or hd-UDCA with their profiles at entry to identify time-associated miRNA changes within each group. The post-entry profiles were assigned to one of two groups: Year 1–4 (Late A), and Year 5 (Late B). Both groups experienced an increase in the number of differentially expressed miRNAs (DE-miRNAs) over time, but the change was more pronounced in the hd-UDCA group. In the placebo group, the number of DE-miRNAs increased from four at Late A (Year 1–4) to 21 at Late B (Year 5) (Fig. 1A). By comparison, the number of DE-miRNAs in the hd-UDCA group increased from 16 in Late A to 42 in Late B (Fig. 1A). Overall, 65 DE-miRNAs showed significant concentration changes (fold change >1.5, *p* <0.05) in at least one post-entry time point in either the placebo or hd-UDCA group (Table S1). We used an unsupervised hierarchical cluster to illustrate the changes in these 65 miRNAs in both the placebo and hd-UDCA groups. All groups from two post-entry time points were clustered according to treatment, suggesting that the patients treated with placebo and those treated with hd-UDCA had distinct serum miRNA profiles (Fig. 1B). In contrast to miRNA, we did not observe significant time-associated concentration changes for the 27 cytokines measured in serum.

The changes in DE-miRNAs between the two time points within the patient groups were also examined. Only one miRNA, miR-92b, showed an increased concentration at both time points (Fig. 1C), while the levels of seven miRNAs (miR-100, miR-122, miR-125b, miR-194, miR-215, miR-375, and miR-99a) decreased at both time points within the hd-UDCA group. There was no common DE-miRNA between the two time points within the placebo group (Fig. 1D).

### hd-UDCA treatment altered serum miRNA and cytokine profiles in patients with PSC

**Fig. 2:**
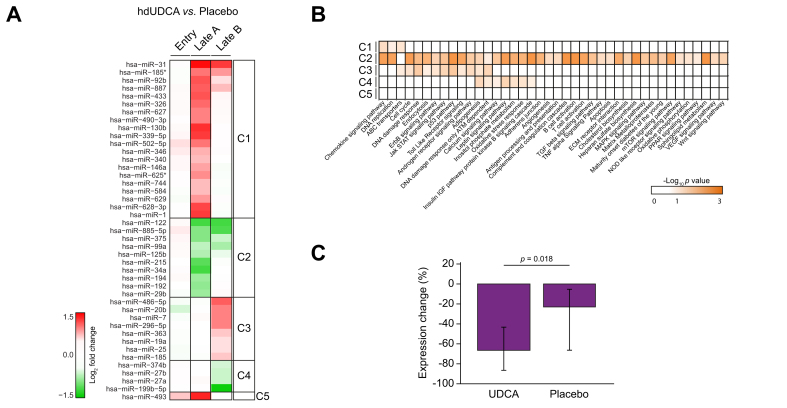
hd-UDCA treatment alters the levels of a group of miRNAs and the cytokine TRAIL in serum. (A) Heat map of the changes in concentration of serum miRNA signatures in the hd-UDCA patient group compared with the placebo group at three different time points (entry, Late A, and Late B). The miRNAs can be grouped in five different clusters (C1–C5) based on the changes in concentration at different time points. (B) Heat map of the significance of pathways enriched by DE-miRNAs in each cluster. The scale indicates –log_10_ transformed *p* values, with the darker colour indicating greater significance. Level of significance: *p* <0.05, Fisher’s exact test. (C) Serum concentration of the TRAIL cytokine between patients treated with hd-UDCA and those treated with placebo. The percentage change represents the expression level change at post-entry time points relative to the baseline level at entry. Data are presented as mean ± SD. Level of significance: *p* <0.05, Student’s *t* test. DE, differentially expressed; hd-UDCA, high-dose ursodeoxycholic acid.

Next, we compared the post-entry serum miRNA profiles between patients treated with hd-UDCA and the placebo group to identify the impact of hd-UDCA treatment on the serum miRNA profile. Based on the same selection criteria used above, 43 DE-miRNAs were identified (Table S2), which grouped into five patterns (C1–C5; Fig. 2A). The C1 and C2 clusters were miRNAs affected only at the Late A time point, which comprised 20 miRNAs with increased concentrations and 10 miRNAs with decreased concentrations. The C3 and C4 clusters were miRNAs affected at the Late B time point, which comprised eight miRNAs with increased concentrations and four with decreased concentrations. The C5 cluster contained only one miRNA, miR-493, which showed an increased concentration between entry and Late A. Although there were no miRNAs showing statistically significant concentration changes between the two consecutive time points, some miRNAs showed the same trend in concentration change at both time points. For example, miR-31, miR-185∗, miR-92b, and miR-887 showed a >1.5-fold change at both time points even though *p* >0.05 at Late B. Interestingly, miR-31 has been documented to be involved in the pathogenesis of liver fibrosis,^20^ and it has been shown that miR-92b promotes HCC progression.^21^ In addition, the concentrations of miR-122, miR-885-5p, miR-375, and miR-99a showed a >1.5-fold decrease at both time points. Notably, miR-122, a liver-enriched miRNA involved in liver homeostasis, demonstrated the greatest concentration decrease upon hd-UDCA treatment at Late A timepoint (fold change = 0.43, *p* = 5.25E-03). Recently, the serum miR-885-5p level was reported as a potential biomarker to detect liver pathologies, including HCC, liver cirrhosis, and chronic hepatitis B.^22^

To investigate the biological processes represented by DE-miRNAs observed in hd-UDCA, pathway enrichment analysis was performed for miRNAs in each cluster by using the miEAA tool (Table S3).^18^ MiRNAs in clusters C1 and C3 were associated with ABC transporters and cell cycle-related pathways (*e.g.* DNA replication in C1 and cell cycle and DNA damage response in C3) (Fig. 2B). Clusters C2 and C4 showed decreased concentrations in miRNAs associated with adipogenesis, ATM-dependent DNA damage response, calcium signaling pathways, leptin signaling pathway, inositol phosphate metabolism, oxidative stress response, and the insulin–IGF pathway protein kinase B signaling cascade. Cluster C2 miRNAs were also associated with several immune/inflammation-related responses [*e.g.* antigen processing and presentation, complement and coagulation cascades, B/T cell activation, transforming growth factor (TGF)-β signaling pathways, etc.], which may represent the involvement of inflammation in PSC pathogenesis. Taken together, these data show that hd-UDCA treatment markedly impacted the serum miRNA profiles in patients with PSC and suggest that UDCA promoted pathways leading to liver-related adverse events.

We also compared the serum cytokine profiles between patients treated with hd-UDCA and the placebo group, and identified only one cytokine with a significant concentration difference between the two groups. The serum level of TRAIL decreased in both groups post-entry, but showed a greater reduction in the hd-UDCA group [mean = –76% (–84.2% to –61.9%)] compared with the placebo group [mean = –31.4% (–65.1% to –4.7%)], with *p* = 0.018 (Fig. 2C).

### Serum miRNA profile may associate with SAEs

**Fig. 3:**
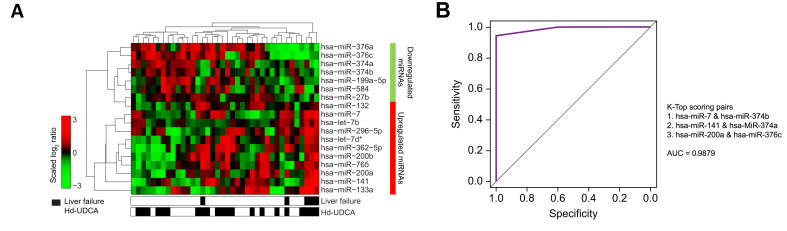
Serum miRNA signatures associated with treatment outcome. (A) Unsupervised hierarchical cluster demonstrating the concentration differences of liver failure-associated miRNA signatures in 37 patients at post-entry time points. Red represents increased concentrations; green represents decreased concentrations. (B) The ROC curve indicates the sensitivity and specificity of three top-scoring miRNA pairs as a signature to identify patients with liver failure after hd-UDCA treatment. hd-UDCA, high-dose ursodeoxycholic acid; ROC, receiver operating characteristic.

We also investigated whether there were specific circulating miRNAs associated with the SAEs observed in the study. Increased serum concentrations of miR-339-5p and miR-200a, and decreased levels of miR-188-5p, were observed in patients who developed EVs (Table 4), compared with patients without EV. Increased concentrations of miR-133a, miR-124, and let-7e were seen in patients who developed CC (Table 4), compared with patients without CC. Notably, patients who progressed to liver failure resulting in liver-related death or liver transplantation demonstrated a unique miRNA pattern with 18 DE-miRNAs (11 showed increased concentrations and seven showed decreased concentrations) (Table 4; Fig. 3A). Among the affected miRNAs, the levels of miR-133a (3.13-fold, *p* = 1.45E-02), miR-141 (2.70-fold, *p* = 8.94E-03), miR-200a (2.59-fold, *p* = 1.61E-03), miR-200b (2.67-fold, *p* = 1.00E-02), miR-296-5p (2.08-fold, *p* = 1.63E-02), miR-362-5p (2.31-fold, *p* = 1.14E-02), and miR-7 (2.53-fold, *p* = 8.54E-04) increased more than twofold, while the levels of miR-376a (0.41-folds, p=2.09E-02) and miR-376c (0.41-folds, p=2.05E-02) decreased the most.

**Table 4:** **Serum miRNAs associated with serious adverse events after hd-UDCA treatment**.

Serious adverse event	miRNA	logFC	*p* value∗
Esophageal varices	hsa-miR-200a	0.67	4.85E-02
	hsa-miR-339-5p	0.65	4.75E-02
	hsa-miR-188-5p	–0.61	3.95E-02
Cholangiocarcinoma	hsa-let-7e	0.93	2.27E-02
	hsa-miR-124	1.78	2.16E-02
	hsa-miR-133a	1.71	2.17E-02
	hsa-miR-502-5p	–1.32	2.20E-02
Liver failure	hsa-let-7b	0.71	2.97E-02
	hsa-let-7d-star	0.83	2.50E-02
	hsa-miR-132	0.62	2.70E-02
	hsa-miR-133a	1.65	1.45E-02
	hsa-miR-141	1.43	8.94E-02
	hsa-mir-200a	1.37	1.61E-02
	hsa-mir-200b	1.42	1.00E-02
	hsa-mir-296-5p	1.06	1.63E-02
	hsa-mir-362-5p	1.21	1.14E-02
	hsa-mir-7	1.34	8.54E-02
	hsa-mir-765	0.78	4.94E-02
	hsa-mir-199a-5p	–0.73	4.85E-02
	hsa-mir-27b	–0.59	3.02E-02
	hsa-mir-374a	–0.88	1.26E-02
	hsa-mir-374b	–0.73	1.40E-02
	hsa-mir-376a	–1.29	2.09E-02
	hsa-mir-376c	–1.27	2.05E-02
	hsa-mir-584	–0.85	1.48E-02

∗ Level of significance: *p* <0.05 (moderated *t* test by LIMMA).

The ability of affected miRNAs to predict liver failure was also evaluated. Three top-scoring pairs of six miRNAs were identified that can predict liver failure (AUC = 0.9879; Fig. 3B). However, due to the small sample size (n = 5), this observation needs to be verified with a larger sample.

**Fig. 4:**
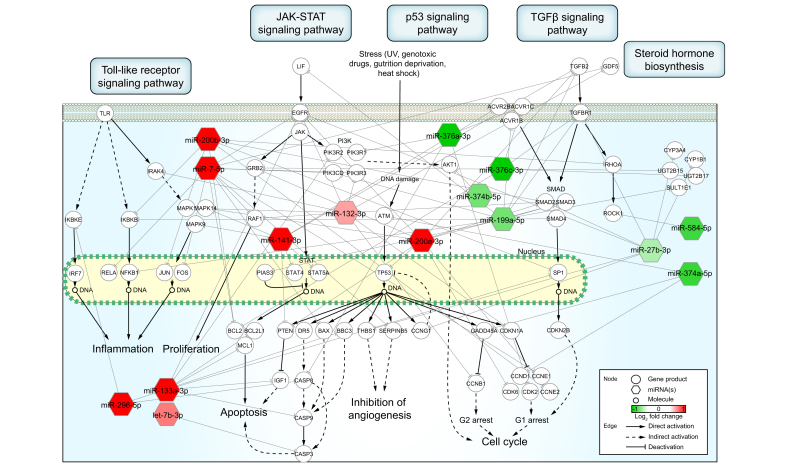
Signaling pathways affected by miRNA changes associated with hd-UDCA treatment; potential mechanisms for liver-related adverse events. KEGG pathways were identified by pathway enrichment analysis using serum miRNAs altered in liver failure. Five key pathways were selected and highlighted in the network. The serum miRNAs were connected to their target genes in the pathways using miRTarBase, which is an experimentally validated interaction database. Circles and hexagons represent gene products and miRNAs, respectively. Red and green hexagons indicate up- and downregulation of miRNA in samples with liver failure, respectively. Solid and dashed arrows show direct and indirect interaction, respectively. Arrowheads or blocked lines denote activation or inhibition, respectively. DE, differentially expressed; hd-UDCA, high-dose ursodeoxycholic acid; KEGG, Kyoto Encyclopedia of Genes and Genomes; TGF, transforming growth factor.

To investigate the biological pathways associated with DE-miRNAs identified in serum samples of patients who later developed liver failure, pathway enrichment analysis was performed (Table S4). Based on the enriched pathways and the miRNA–target interactions (obtained from miRTarBase version 7.0)^23^ (Table S5), we constructed a perturbed hypothetical network in patients who later developed liver failure reflected by their altered serum miRNA signature (Fig. 4). The network analysis revealed that miRNAs that showed increased concentration were involved in cell survival responses, such as inflammation (*e.g.* JAK-STAT and NF-κB), proliferation (*e.g.* GRB2 and RAF1), and apoptosis (*e.g.* BCL2, BAX, and CASP3/8/9) and miRNAs with decreased concentrations were involved in the cell cycle (*e.g.* CDK2/8, CDKN1A, and CKDN2B), fibrosis (*e.g.* TGFB2, TGFBR1, and SMAD2/3/4), and sterol biosynthesis (*e.g.* UGT2B15 and UGT2B17).

## Discussion

It has been previously recognised that patients with PSC have distinct miRNAs in their serum and bile,^24^ although these have not been evaluated longitudinally or in the context of ursodiol therapy. Our study provides unique insight into miRNA serum profiles in patients with PSC and demonstrates changes over time observed with hd-UDCA treatment. To our knowledge, this is the only report of longitudinal changes in miRNA profiles in patients with PSC and in those treated with hd-UDCA compared with placebo.

Our data showed hd-UDCA treatment-related changes in miRNAs associated with immune-mediated and inflammation-related responses (*e.g.* antigen processing and presentation, complement and coagulation cascades, B/T cell activation, TGFβ signaling pathways, etc.). The alteration in miRNA and cytokine profiles in PSC identified in our cohort may provide a useful platform for further research of these markers in understanding disease pathogenesis in PSC.

In addition, we were able to analyse changes in miRNA patterns in patients receiving either placebo or hd-UDCA, both at specific time points and longitudinally. When analysing only the placebo-treated group at early *vs.* late time points, we found an increased number of differentially expressed miRNAs, suggesting that serum miRNA profiles evolve during the natural course of the disease. Moreover, a larger proportion of differentially expressed miRNAs was identified in patients treated with hd-UDCA, Among these miRNAs, only the concentration of miR-92b increased with hd-UDCA treatment, which, interestingly, has previously been shown to be an oncogene,^25^ specifically via inhibition of cell cycle checkpoint gene *p57*.^26^ Of the seven miRNAs the concentrations of which decreased, miR-885-5p, miR-122, and miR-194 have previously been observed to be upregulated in PSC and CC, as well as in other liver diseases.^27^ This finding may again suggest a tumor suppressor role for these miRNAs via CDK2 and MCM5,^28^ which is enhanced early in PSC progression and may become exhausted later in disease or when the condition is exacerbated by hd-UDCA treatment. miR-375, another miRNA with a decreased concentration in all patients treated with UDCA, has previously been found to be downregulated in fibrolamellar carcinoma.^29^ miR-99a and miR-125b are known to be downregulated in CC^30^ and are on the same cluster on chromosome 21q21.^31^ Further mechanistic analysis would need to confirm which of these candidates has a key role in PSC pathogenesis, paying close attention to the temporal onset and magnitude of concentration changes to determine the overall diagnostic or prognostic value of these serum miRNAs. Overall, our findings highlight that, along with the previous description of increased disease severity and development of portal hypertension, unique differential changes in miRNA were observed in patients treated with hd-UDCA.

A better understanding of the relationship between changes in serum miRNA profiles and clinical course of PSC may contribute to a better understanding of disease mechanisms and aid the development of therapies for this liver disease. Increased neoplasia was previously observed among patients treated with hd-UDCA, suggesting that the latter induces dysregulation of cell proliferation or cell death.^32^ Interestingly, this observation was consistent with the hd-UDCA treatment-associated serum miRNA patterns identified in this study (Fig. 2A,B). In the hd-UDCA group, we observed decreased levels of antiproliferation miRNAs, such as miR-125b.^33^ Furthermore, miR-122 concentration in serum was significantly decreased in the hd-UDCA group compared with the placebo-group (Fig. 2A,B); an increased miR-122 level in blood may indicate increased liver injury.^34^ There are unique opportunities through the study and utilisation of miRNA for prognostication and therapeutic intervention in hepatobiliary disease,^35^ and our work further enhances the need for more in-depth study of miRNAs in PSC.

Additional strengths of our study are that it was derived from the only randomised control trial evaluating hd-UDCA *vs.* placebo in patients with PSC, and the only longitudinal data set evaluating miRNA changes in these patients. Limitations of this study include the relatively small sample size and lack of individual patient data; we hope that these findings may serve as a stepping-stone for a larger clinical trial that may further validate our findings, bringing cell-free blood miRNA biomarkers closer to clinical applications.

We also acknowledge that the spectrum of cell-free circulating miRNA, similar to all other biomolecules in the blood, such as proteins, is affected by many factors, including treatment as well as disease state. This is the foundation of using the changes of certain blood biomolecules, including specific miRNA species, as biomarkers for disease diagnosis, therapeutic efficacy, and others. Biological and technical factors can also affect the spectrum of cell-free miRNA. These include gender, age, blood sampling time, ample preparation methods, storage conditions, measurement methods, and others, which is why we previously highlighted the importance of standardisation for analysing cell-free RNA.^36^ Nevertheless, the miRNA changes identified in this study were determined using the same measurement and sample preparation methods. Even though other factors might affect the spectrum of circulating miRNA, the major contribution factors are the disease state and treatment difference.

In conclusion, we identified unique longitudinal changes in serum miRNA profiles in patients with PSC and alterations of several miRNA concentrations in patients treated with hd-UDCA. Our study shows that hd-UDCA treatment contributes to a unique alteration in miRNA in patients with PSC that is distinctly different at both cross-sectional and longitudinal time points. Further investigation is warranted to determine whether these changes in miRNA concentrations are directly caused by treatment with hd-UDCA, which in turn then causes increased liver-related adverse events, or whether the hd-UDCA may be contributing to direct liver injury secondary to bile acid toxicity. Future studies examining miRNA changes in patients treated with standard-dose UDCA are also needed, because recent guidelines have endorsed its use.^37^

## Financial support

This study was supported by 10.13039/100000002National Institutes of Health grants 3R01DK056924-08S1, 5K24DK002957, and 1R21HL112678.

## Authors’ contributions

JTH, AS, and EG reviewed the literature and drafted the article. PH, BDM, T-KK, KW, YL, JBR, and JEN were involved in performing the experiments and statistical analysis for the study. KDL, NFL, and KVK designed the study, revised the manuscript for intellectual content, and were involved in the final approval of the publication. All authors agree to be accountable for their aspects of the work.

## Data availability statement

The data that support the findings of this study are available from the corresponding author, KVK, upon reasonable request. The design of the study has been previously published and the information is provided in the Materials and Methods of that publication.

## Conflicts of interest

The authors declare no conflicts of interest that pertain to this work.

Please refer to the accompanying ICMJE disclosure forms for further details.
