# Supplementary material for: Serum miRNA profiles are altered in patients with primary sclerosing cholangitis receiving high-dose ursodeoxycholic acid
Source: JHEP Rep. 2023 Mar 23;5(6):100729. doi: 10.1016/j.jhepr.2023.100729 (PMC10172698; doi:10.1016/j.jhepr.2023.100729)
Supplement: Multimedia component 1 [file mmc1.pdf]

Serum miRNA profiles are altered in patients with primary sclerosing cholangitis receiving high-dose ursodeoxycholic acid

Jessica T. Hochberg, Aalam Sohal, Priya Handa, Bryan D.Maliken, Take-Kyun Kim, Kai Wang, Eric Gochanour, Yu Li, J Bart Rose, James E. Nelson[4], Keith D Lindor, Nicholas F. LaRusso, Kris V.Kowdley

Table of contents

1. Table S1
2. Table S2
3. Table S3
4. Table S4
5. Table S5

Table S1. Differentially expressed miRNAs at later time points in placebo or hd-UDCA

miRNA	LateA vs Placebo		LateB vs Placebo		LateA vs hd-UDCA		LateB vs hd-UDCA	
	logFC	p-Value	logFC	p-Value	logFC	p-Value	logFC	p-Value
hsa-let-7a	0.24	0.28	0.87	0.00	0.45	0.07	0.50	0.03
hsa-let-7d	0.34	0.16	0.83	0.00	0.43	0.06	0.70	0.00
hsa-let-7d*	-0.68	0.01	-0.40	0.17	-0.20	0.51	-0.13	0.67
hsa-let-7e	0.61	0.11	0.85	0.03	0.45	0.17	-0.13	0.70
hsa-let-7f	0.27	0.33	0.93	0.00	0.24	0.37	0.78	0.01
hsa-let-7i	0.10	0.67	0.39	0.09	0.17	0.40	0.88	0.00
hsa-miR-100	0.21	0.55	-0.24	0.55	-0.80	0.04	-1.15	0.01
hsa-miR-122	0.50	0.19	-0.19	0.67	-1.00	0.02	-1.67	0.00
hsa-miR-125b	0.27	0.34	-0.57	0.06	-0.68	0.01	-1.26	0.00
hsa-miR-142-3p	0.14	0.61	0.70	0.02	0.51	0.06	0.35	0.19
hsa-miR-146b-5p	-0.08	0.74	-0.19	0.46	-0.03	0.91	-0.62	0.01
hsa-miR-148a	-0.06	0.79	-0.37	0.12	-0.57	0.04	-1.06	0.00
hsa-miR-150	-0.02	0.95	-0.40	0.20	-0.42	0.09	-0.70	0.01
hsa-miR-152	-0.19	0.38	-0.23	0.30	-0.37	0.13	-0.71	0.01
hsa-miR-16	-0.08	0.71	-0.05	0.83	-0.03	0.91	0.74	0.01
hsa-miR-17	0.17	0.57	0.05	0.88	0.13	0.60	0.62	0.02
hsa-miR-182	-0.12	0.73	0.49	0.18	0.46	0.12	0.65	0.03
hsa-miR-185	0.04	0.86	-0.09	0.74	0.15	0.63	0.88	0.01
hsa-miR-18b	0.09	0.70	-0.24	0.33	0.39	0.12	0.62	0.02
hsa-miR-191	0.25	0.21	0.60	0.01	0.21	0.32	0.34	0.12
hsa-miR-192	0.36	0.32	-0.93	0.01	-0.53	0.15	-0.83	0.04
hsa-miR-193a-5p	0.11	0.68	-0.12	0.70	-0.45	0.23	-1.07	0.00
hsa-miR-193b	0.15	0.71	-0.51	0.22	-0.42	0.38	-1.09	0.04
hsa-miR-194	0.14	0.62	-0.54	0.09	-0.99	0.00	-0.88	0.02
hsa-miR-199b-5p	-0.26	0.52	1.11	0.01	-0.13	0.70	-0.32	0.40
hsa-miR-19a	-0.02	0.93	-0.08	0.72	0.22	0.25	0.75	0.00
hsa-miR-20a	0.08	0.69	-0.10	0.65	0.03	0.90	0.61	0.01
hsa-miR-20b	-0.40	0.22	-0.51	0.14	0.15	0.72	1.02	0.02
hsa-miR-214	-0.34	0.37	-0.81	0.05	-0.09	0.82	-0.65	0.10
hsa-miR-215	0.34	0.34	-0.68	0.07	-0.78	0.04	-0.92	0.04
hsa-miR-221	0.24	0.33	0.67	0.01	0.44	0.12	0.22	0.47
hsa-miR-23b	-0.11	0.52	0.03	0.84	-0.11	0.58	-0.64	0.00
hsa-miR-25	-0.12	0.55	-0.16	0.45	0.12	0.64	0.78	0.00
hsa-miR-26a	0.06	0.81	0.74	0.00	0.14	0.51	0.11	0.60
hsa-miR-26b	0.15	0.39	0.60	0.00	-0.08	0.69	0.30	0.17
hsa-miR-27b	0.02	0.92	-0.14	0.53	-0.04	0.84	-0.85	0.00
hsa-miR-296-5p	-0.18	0.60	-0.71	0.04	-0.09	0.74	0.32	0.21
hsa-miR-29a	0.18	0.58	-0.36	0.25	-0.39	0.11	-0.93	0.00
hsa-miR-30a	0.14	0.68	-0.34	0.37	-0.50	0.14	-0.85	0.02
hsa-miR-31	-0.31	0.43	-1.60	0.00	0.95	0.03	-0.54	0.23
hsa-miR-329	-0.14	0.70	-0.34	0.38	0.76	0.03	-0.23	0.52
hsa-miR-339-5p	0.03	0.93	0.70	0.11	0.86	0.01	0.36	0.30
hsa-miR-33a	0.11	0.71	0.68	0.04	0.41	0.16	0.40	0.18
hsa-miR-345	-0.05	0.83	-0.82	0.00	0.19	0.46	-0.89	0.00
hsa-miR-34a	1.07	0.02	0.02	0.97	-0.33	0.48	-0.17	0.72
hsa-miR-373-star	-0.51	0.30	-1.89	0.00	0.63	0.14	-1.05	0.01
hsa-miR-374b	0.46	0.12	1.03	0.00	0.35	0.21	0.29	0.30
hsa-miR-375	0.29	0.59	-0.17	0.76	-1.08	0.01	-1.31	0.02

hsa-miR-378	0.00	0.99	-0.31	0.17	-0.50	0.05	-0.76	0.01
hsa-miR-423-3p	0.28	0.17	0.49	0.03	0.59	0.00	0.10	0.61
hsa-miR-424	-0.69	0.01	-0.22	0.37	-0.19	0.38	-0.48	0.02
hsa-miR-433	-0.07	0.85	-0.15	0.71	0.89	0.01	0.15	0.66
hsa-miR-486-5p	-0.09	0.77	-0.06	0.83	0.23	0.46	1.46	0.00
hsa-miR-513a-5p	-0.01	0.98	-0.57	0.15	0.40	0.30	-0.79	0.04
hsa-miR-574-3p	0.43	0.09	-0.42	0.11	-0.19	0.50	-0.68	0.02
hsa-miR-584	0.04	0.91	0.56	0.11	0.85	0.01	0.42	0.19
hsa-miR-589	0.23	0.51	-1.02	0.01	0.94	0.01	-0.62	0.11
hsa-miR-598	-0.29	0.40	-0.07	0.84	-0.48	0.14	-0.65	0.02
hsa-miR-622	-0.58	0.06	-1.13	0.01	0.09	0.82	-1.15	0.02
hsa-miR-628-3p	-0.85	0.01	0.20	0.53	0.42	0.20	0.04	0.90
hsa-miR-629	-0.58	0.03	-0.13	0.64	0.70	0.02	0.42	0.15
hsa-miR-663	-0.25	0.45	-1.16	0.00	0.40	0.26	-0.46	0.17
hsa-miR-7	-0.12	0.75	-0.13	0.71	0.40	0.22	1.10	0.00
hsa-miR-885-5p	0.45	0.33	-0.16	0.75	-1.01	0.05	-1.85	0.00
hsa-miR-92b	-0.58	0.04	0.31	0.28	0.68	0.03	1.03	0.00
hsa-miR-93	0.17	0.43	-0.09	0.68	0.10	0.66	0.61	0.01
hsa-miR-99a	0.01	0.96	-0.55	0.10	-0.93	0.01	-1.61	0.00

Table S2. Differentially expressed miRNAs in hd-UDCA compared to Placebo

miRNA	hd-UDCA vs Placebo at Entry		hd-UDCA vs Placebo at Late A		hd-UDCA vs Placebo at Late B		Cluster
	logFC	p-Value	logFC	p-Value	logFC	p-Value	Number
hsa-miR-31	0.18	0.54	1.44	0.00	1.23	0.06	C1
hsa-miR-122	0.28	0.34	-1.21	0.01	-1.20	0.08	C2
hsa-miR-125b	0.22	0.27	-0.73	0.01	-0.47	0.19	C2
hsa-miR-493	0.67	0.04	1.31	0.01	0.10	0.83	C5
hsa-miR-130b	0.32	0.04	1.28	0.00	0.05	0.83	C1
hsa-miR-185	-0.29	0.16	-0.19	0.50	0.68	0.03	C3
hsa-miR-1	-0.09	0.78	1.26	0.02	-0.26	0.73	C1
hsa-miR-192	0.02	0.95	-0.87	0.03	0.11	0.80	C2
hsa-miR-194	0.30	0.16	-0.83	0.02	-0.04	0.93	C2
hsa-miR-199b-5p	-0.02	0.92	0.10	0.81	-1.45	0.00	C4
hsa-miR-19a	-0.23	0.09	0.01	0.97	0.60	0.04	C3
hsa-miR-20b	-0.57	0.04	-0.02	0.96	0.96	0.00	C3
hsa-miR-215	0.04	0.88	-1.08	0.01	-0.20	0.73	C2
hsa-miR-25	-0.33	0.05	-0.09	0.71	0.60	0.01	C3
hsa-miR-27a	0.05	0.69	0.24	0.33	-0.63	0.03	C4
hsa-miR-27b	0.08	0.58	0.02	0.93	-0.63	0.02	C4
hsa-miR-296-5p	-0.05	0.81	0.04	0.92	0.98	0.01	C3
hsa-miR-29b	-0.26	0.15	-0.83	0.03	0.30	0.14	C2
hsa-miR-339-5p	0.42	0.11	1.25	0.00	0.08	0.83	C1
hsa-miR-628-3p	-0.07	0.74	1.20	0.00	-0.23	0.44	C1
hsa-miR-433	0.19	0.45	1.16	0.01	0.49	0.16	C1
hsa-miR-92b	-0.13	0.51	1.13	0.01	0.59	0.05	C1
hsa-miR-887	0.32	0.20	1.10	0.01	0.72	0.11	C1
hsa-miR-34a	0.19	0.56	-1.21	0.02	0.00	1.00	C2
hsa-miR-363	-0.11	0.56	0.04	0.87	0.69	0.02	C3
hsa-miR-374b	0.14	0.48	0.03	0.92	-0.60	0.03	C4
hsa-miR-375	0.48	0.18	-0.89	0.03	-0.65	0.36	C2
hsa-miR-326	0.28	0.18	1.05	0.02	0.37	0.26	C1
hsa-miR-486-5p	-0.42	0.04	-0.11	0.76	1.10	0.00	C3
hsa-miR-185-star	-0.17	0.60	1.03	0.04	0.86	0.06	C1
hsa-miR-502-5p	0.58	0.02	1.01	0.01	0.14	0.82	C1
hsa-miR-627	0.24	0.29	0.98	0.01	0.24	0.50	C1
hsa-miR-490-3p	0.13	0.62	0.97	0.02	0.20	0.61	C1
hsa-miR-625*	0.12	0.59	0.89	0.03	-0.24	0.62	C1
hsa-miR-629	-0.39	0.04	0.89	0.02	0.16	0.65	C1
hsa-miR-146a	0.40	0.02	0.83	0.00	-0.42	0.17	C1
hsa-miR-346	0.53	0.13	0.76	0.04	0.16	0.85	C1
hsa-miR-7	-0.27	0.27	0.25	0.55	0.96	0.01	C3
hsa-miR-744	0.07	0.67	0.73	0.01	-0.12	0.72	C1
hsa-miR-885-5p	0.52	0.15	-0.93	0.04	-1.17	0.11	C2
hsa-miR-340	0.42	0.04	0.72	0.03	0.01	0.97	C1
hsa-miR-584	-0.10	0.65	0.71	0.03	-0.24	0.52	C1
hsa-miR-99a	0.28	0.24	-0.67	0.03	-0.78	0.06	C2

Table S3- Table S3. Pathways enriched by differentially expressed miRNAs in hd-UDCA compared to placebo. Mann-whiteny test was used to compare groups.

		p-Value					miRNA				
Pathway id	Pathway	C1	C2	C3	C4	C5	C1	C2	C3	C4	C5
hsa04062	Chemokine signaling pathway	0.02	0	1	1	1	hsa-miR-1-3p; hsa-miR-130b-3p; hsa-miR-146a-5p; hsa-miR-31-5p; hsa-miR-326; hsa-miR-339-5p; hsa-miR-346; hsa-miR-433-3p; hsa-miR-584-5p; hsa-miR-744-5p; hsa-miR-92b-3p	hsa-miR-122-5p; hsa-miR-125b-5p; hsa-miR-192-5p; hsa-miR-194-5p; hsa-miR-215-5p; hsa-miR-29b-3p; hsa-miR-34a-5p; hsa-miR-99a-5p			
hsa03030	DNA replication	0.05	0	1	1	1	hsa-miR-1-3p; hsa-miR-130b-3p; hsa-miR-31-5p; hsa-miR-339-5p; hsa-miR-744-5p	hsa-miR-125b-5p; hsa-miR-192-5p; hsa-miR-215-5p; hsa-miR-34a-5p; hsa-miR-885-5p			
hsa02010	ABC transporters	0.03	1	0.04	1	1	hsa-miR-1-3p; hsa-miR-339-5p; hsa-miR-346; hsa-miR-433-3p; hsa-miR-92b-3p		hsa-miR-19a-3p; hsa-miR-296-5p; hsa-miR-7-5p		
hsa04110	Cell cycle	1	0	0.03	1	1		hsa-miR-122-5p; hsa-miR-125b-5p; hsa-miR-192-5p; hsa-miR-194-5p; hsa-miR-215-5p; hsa-miR-29b-3p; hsa-miR-34a-5p; hsa-miR-885-5p; hsa-miR-99a-5p	hsa-miR-185-5p; hsa-miR-19a-3p; hsa-miR-20b-5p; hsa-miR-25-3p; hsa-miR-296-5p; hsa-miR-363-3p; hsa-miR-7-5p		
WP707	DNA damage response	1	0	0.01	1	1		hsa-miR-122-5p; hsa-miR-125b-5p; hsa-miR-192-5p; hsa-miR-215-5p; hsa-miR-29b-3p; hsa-miR-34a-5p;	hsa-miR-185-5p; hsa-miR-19a-3p; hsa-miR-20b-5p; hsa-miR-25-3p; hsa-miR-296-5p;		

								hsa-miR-885-5p; hsa-miR-99a-5p	hsa-miR-363-3p; hsa-miR-7-5p		
hsa04144	Endocytosis	1	0	0.05	1	1		hsa-miR-122-5p; hsa-miR-125b-5p; hsa-miR-192-5p; hsa-miR-194-5p; hsa-miR-215-5p; hsa-miR-29b-3p; hsa-miR-34a-5p; hsa-miR-99a-5p	hsa-miR-185-5p; hsa-miR-19a-3p; hsa-miR-20b-5p; hsa-miR-25-3p; hsa-miR-296-5p; hsa-miR-7-5p		
hsa04012	ErbB signaling pathway	1	0.01	0.03	1	1		hsa-miR-122-5p; hsa-miR-125b-5p; hsa-miR-192-5p; hsa-miR-194-5p; hsa-miR-215-5p; hsa-miR-34a-5p; hsa-miR-99a-5p	hsa-miR-185-5p; hsa-miR-19a-3p; hsa-miR-20b-5p; hsa-miR-296-5p; hsa-miR-363-3p; hsa-miR-7-5p		
hsa04630	Jak STAT signaling pathway	1	0.01	0.02	1	1		hsa-miR-122-5p; hsa-miR-125b-5p; hsa-miR-192-5p; hsa-miR-194-5p; hsa-miR-215-5p; hsa-miR-29b-3p; hsa-miR-34a-5p	hsa-miR-185-5p; hsa-miR-19a-3p; hsa-miR-20b-5p; hsa-miR-25-3p; hsa-miR-296-5p; hsa-miR-7-5p		
P00059	p53 pathway	1	0	0.01	1	1		hsa-miR-122-5p; hsa-miR-125b-5p; hsa-miR-192-5p; hsa-miR-194-5p; hsa-miR-215-5p; hsa-miR-29b-3p; hsa-miR-34a-5p; hsa-miR-885-5p; hsa-miR-99a-5p	hsa-miR-185-5p; hsa-miR-19a-3p; hsa-miR-20b-5p; hsa-miR-25-3p; hsa-miR-296-5p; hsa-miR-363-3p; hsa-miR-7-5p		
WP75	Toll Like Receptor signaling	1	0	0.04	1	1		hsa-miR-122-5p; hsa-miR-125b-5p; hsa-miR-192-5p; hsa-miR-194-5p; hsa-miR-215-5p; hsa-miR-29b-3p; hsa-miR-34a-5p	hsa-miR-185-5p; hsa-miR-19a-3p; hsa-miR-296-5p; hsa-miR-486-5p; hsa-miR-7-5p		
WP138	Androgen receptor signaling pathway	1	0.01	0.02	1	1		hsa-miR-122-5p; hsa-miR-125b-5p; hsa-miR-192-5p; hsa-miR-	hsa-miR-185-5p; hsa-miR-19a-3p; hsa-miR-20b-5p;		

								194-5p; hsa-miR-215-5p; hsa-miR-29b-3p; hsa-miR-34a-5p; hsa-miR-99a-5p	hsa-miR-25-3p; hsa-miR-296-5p; hsa-miR-363-3p; hsa-miR-7-5p		
WP236	Adipogenesis	1	0.01	0.03	0.02	1		hsa-miR-122-5p; hsa-miR-125b-5p; hsa-miR-192-5p; hsa-miR-215-5p; hsa-miR-29b-3p; hsa-miR-34a-5p; hsa-miR-99a-5p	hsa-miR-185-5p; hsa-miR-19a-3p; hsa-miR-20b-5p; hsa-miR-25-3p; hsa-miR-363-3p; hsa-miR-7-5p	hsa-miR-199b-5p; hsa-miR-27a-3p; hsa-miR-27b-3p; hsa-miR-374b-5p	
WP710	DNA damage response only ATM dependent	1	0.05	0.02	0.04	1		hsa-miR-122-5p; hsa-miR-125b-5p; hsa-miR-192-5p; hsa-miR-194-5p; hsa-miR-215-5p; hsa-miR-29b-3p; hsa-miR-34a-5p	hsa-miR-185-5p; hsa-miR-19a-3p; hsa-miR-20b-5p; hsa-miR-25-3p; hsa-miR-296-5p; hsa-miR-363-3p; hsa-miR-7-5p	hsa-miR-199b-5p; hsa-miR-27a-3p; hsa-miR-27b-3p; hsa-miR-374b-5p	
hsa04020	Calcium signaling pathway	1	0.01	1	0.05	1		hsa-miR-122-5p; hsa-miR-125b-5p; hsa-miR-192-5p; hsa-miR-215-5p; hsa-miR-34a-5p; hsa-miR-99a-5p		hsa-miR-199b-5p; hsa-miR-27a-3p; hsa-miR-27b-3p	
WP2034	Leptin signaling pathway	1	0	1	0.02	1		hsa-miR-122-5p; hsa-miR-125b-5p; hsa-miR-192-5p; hsa-miR-194-5p; hsa-miR-215-5p; hsa-miR-29b-3p; hsa-miR-34a-5p; hsa-miR-99a-5p		hsa-miR-199b-5p; hsa-miR-27a-3p; hsa-miR-27b-3p; hsa-miR-374b-5p	
hsa00562	Inositol phosphate metabolism	1	0	1	0.03	1		hsa-miR-122-5p; hsa-miR-125b-5p; hsa-miR-192-5p; hsa-miR-215-5p; hsa-miR-29b-3p; hsa-miR-34a-5p		hsa-miR-27a-3p; hsa-miR-27b-3p; hsa-miR-374b-5p	
P00046	Oxidative stress response	1	0	1	0.03	1		hsa-miR-122-5p; hsa-miR-125b-5p; hsa-miR-192-5p; hsa-miR-		hsa-miR-27a-3p; hsa-miR-27b-3p;	

								215-5p; hsa-miR-29b-3p; hsa-miR-34a-5p		hsa-miR-374b-5p	
P00033	Insulin IGF pathway protein kinase B signaling cascade	1	0	1	0.05	1		hsa-miR-122-5p; hsa-miR-125b-5p; hsa-miR-192-5p; hsa-miR-194-5p; hsa-miR-215-5p; hsa-miR-29b-3p; hsa-miR-34a-5p; hsa-miR-99a-5p		hsa-miR-27a-3p; hsa-miR-27b-3p; hsa-miR-374b-5p	
hsa04520	Adherens junction	1	0	1	1	1		hsa-miR-122-5p; hsa-miR-125b-5p; hsa-miR-192-5p; hsa-miR-194-5p; hsa-miR-215-5p; hsa-miR-29b-3p; hsa-miR-34a-5p; hsa-miR-99a-5p			
P00005	Angiogenesis	1	0.02	1	1	1		hsa-miR-122-5p; hsa-miR-125b-5p; hsa-miR-192-5p; hsa-miR-215-5p; hsa-miR-29b-3p; hsa-miR-34a-5p; hsa-miR-99a-5p			
hsa04612	Antigen processing and presentation	1	0.02	1	1	1		hsa-miR-122-5p; hsa-miR-125b-5p; hsa-miR-192-5p; hsa-miR-215-5p; hsa-miR-34a-5p			
hsa04610	Complement and coagulation cascades	1	0	1	1	1		hsa-miR-122-5p; hsa-miR-192-5p; hsa-miR-215-5p; hsa-miR-29b-3p; hsa-miR-99a-5p			
P00010	B cell activation	1	0	1	1	1		hsa-miR-122-5p; hsa-miR-125b-5p; hsa-miR-192-5p; hsa-miR-194-5p; hsa-miR-215-5p; hsa-miR-29b-3p; hsa-miR-34a-5p			

P00053	T cell activation	1	0	1	1	1		hsa-miR-122-5p; hsa-miR-125b-5p; hsa-miR-192-5p; hsa-miR-194-5p; hsa-miR-215-5p; hsa-miR-29b-3p; hsa-miR-34a-5p			
hsa04350	TGF beta signaling pathway	1	0.01	1	1	1		hsa-miR-122-5p; hsa-miR-125b-5p; hsa-miR-192-5p; hsa-miR-194-5p; hsa-miR-215-5p; hsa-miR-29b-3p; hsa-miR-34a-5p			
WP231	TNF alpha Signaling Pathway	1	0.02	1	1	1		hsa-miR-122-5p; hsa-miR-125b-5p; hsa-miR-192-5p; hsa-miR-194-5p; hsa-miR-215-5p; hsa-miR-34a-5p			
hsa04210	Apoptosis	1	0.03	1	1	1		hsa-miR-122-5p; hsa-miR-125b-5p; hsa-miR-192-5p; hsa-miR-215-5p; hsa-miR-29b-3p; hsa-miR-34a-5p			
hsa04512	ECM receptor interaction	1	0	1	1	1		hsa-miR-192-5p; hsa-miR-194-5p; hsa-miR-215-5p; hsa-miR-29b-3p; hsa-miR-34a-5p; hsa-miR-99a-5p			
P00014	Cholesterol biosynthesis	1	0.01	1	1	1		hsa-miR-125b-5p; hsa-miR-192-5p; hsa-miR-215-5p			
hsa00534	Heparan sulfate biosynthesis	1	0	1	1	1		hsa-miR-125b-5p; hsa-miR-192-5p; hsa-miR-215-5p; hsa-miR-34a-5p; hsa-miR-99a-5p			
hsa04010	MAPK signaling pathway	1	0.01	1	1	1		hsa-miR-122-5p; hsa-miR-125b-5p; hsa-miR-192-5p; hsa-miR-			

								194-5p; hsa-miR-215-5p; hsa-miR-29b-3p; hsa-miR-34a-5p; hsa-miR-99a-5p			
WP129	Matrix Metalloproteinases	1	0.01	1	1	1		hsa-miR-125b-5p; hsa-miR-192-5p; hsa-miR-215-5p; hsa-miR-29b-3p			
hsa04950	Maturity onset diabetes of the young	1	0.01	1	1	1		hsa-miR-192-5p; hsa-miR-215-5p; hsa-miR-34a-5p			
hsa04150	mTOR signaling pathway	1	0	1	1	1		hsa-miR-122-5p; hsa-miR-125b-5p; hsa-miR-192-5p; hsa-miR-215-5p; hsa-miR-29b-3p; hsa-miR-34a-5p; hsa-miR-99a-5p			
hsa04621	NOD like receptor signaling pathway	1	0.03	1	1	1		hsa-miR-122-5p; hsa-miR-125b-5p; hsa-miR-192-5p; hsa-miR-215-5p; hsa-miR-34a-5p			
WP623	Oxidative phosphorylation	1	0.03	1	1	1		hsa-miR-125b-5p; hsa-miR-192-5p; hsa-miR-215-5p; hsa-miR-99a-5p			
hsa03320	PPAR signaling pathway	1	0.04	1	1	1		hsa-miR-122-5p; hsa-miR-192-5p; hsa-miR-215-5p; hsa-miR-34a-5p			
hsa00600	Sphingolipid metabolism	1	0	1	1	1		hsa-miR-122-5p; hsa-miR-125b-5p; hsa-miR-192-5p; hsa-miR-215-5p; hsa-miR-34a-5p; hsa-miR-99a-5p			
hsa04370	VEGF signaling pathway	1	0.02	1	1	1		hsa-miR-122-5p; hsa-miR-125b-5p; hsa-			

								miR-194-5p; hsa-miR-29b-3p; hsa-miR-34a-5p; hsa-miR-99a-5p			
hsa04310	Wnt signaling pathway	1	0.02	1	1	1		hsa-miR-122-5p; hsa-miR-125b-5p; hsa-miR-192-5p; hsa-miR-194-5p; hsa-miR-215-5p; hsa-miR-29b-3p; hsa-miR-34a-5p			

Table S4- Pathways enriched by differentially expressed miRNAs in serum of patients who later developed liver failure

KEGG pathway	Up-regulated		Down-regulated	
	p-Value	miRNAs	p-Value	miRNAs
hsa00591 Linoleic acid metabolism	0.03	hsa-let-7b-5p; hsa-miR-132-3p		
hsa02010 ABC transporters	0.03	hsa-let-7b-5p; hsa-miR-132-3p; hsa-miR-296-5p		
hsa04012 ErbB signaling pathway	0.01	hsa-let-7b-5p; hsa-miR-132-3p; hsa-miR-141-3p; hsa-miR-200a-3p; hsa-miR-200b-3p; hsa-miR-296-5p		
hsa04114 Oocyte meiosis	0.04	hsa-let-7b-5p; hsa-miR-132-3p; hsa-miR-141-3p; hsa-miR-200a-3p; hsa-miR-200b-3p		
hsa04115 p53 signaling pathway	0.02	hsa-let-7b-5p; hsa-miR-132-3p; hsa-miR-141-3p; hsa-miR-200a-3p; hsa-miR-200b-3p; hsa-miR-296-5p		
hsa04310 Wnt signaling pathway	0.01	hsa-let-7b-5p; hsa-miR-132-3p; hsa-miR-141-3p; hsa-miR-200a-3p; hsa-miR-200b-3p; hsa-miR-296-5p	0.01	hsa-miR-199a-5p; hsa-miR-27b-3p; hsa-miR-374a-5p; hsa-miR-374b-5p; hsa-miR-584-5p
hsa04350 TGF beta signaling pathway	0.04	hsa-let-7b-5p; hsa-miR-132-3p; hsa-miR-141-3p; hsa-miR-200a-3p; hsa-miR-200b-3p	0.00	hsa-miR-199a-5p; hsa-miR-27b-3p; hsa-miR-374a-5p; hsa-miR-374b-5p; hsa-miR-584-5p
hsa04370 VEGF signaling pathway	0.02	hsa-let-7b-5p; hsa-miR-132-3p; hsa-miR-141-3p; hsa-miR-200a-3p; hsa-miR-200b-3p		
hsa04620 Toll like receptor signaling pathway	0.02	hsa-let-7b-5p; hsa-miR-132-3p; hsa-miR-141-3p; hsa-miR-200a-3p; hsa-miR-296-5p		
hsa04621 NOD like receptor signaling pathway	0.05	hsa-let-7b-5p; hsa-miR-141-3p; hsa-miR-200a-3p; hsa-miR-200b-3p		
hsa04622 RIG I like receptor signaling pathway	0.02	hsa-let-7b-5p; hsa-miR-141-3p; hsa-miR-200a-3p; hsa-miR-296-5p		
hsa04650 Natural killer cell mediated cytotoxicity	0.01	hsa-let-7b-5p; hsa-miR-132-3p; hsa-miR-141-3p; hsa-miR-200a-3p; hsa-miR-200b-3p		
hsa04710 Circadian rhythm mammal	0.04	hsa-let-7b-5p; hsa-miR-141-3p		
hsa04722 Neurotrophin signaling pathway	0.00	hsa-let-7b-5p; hsa-miR-132-3p; hsa-miR-141-3p; hsa-miR-200a-3p; hsa-miR-200b-3p; hsa-miR-296-5p; hsa-miR-765		
hsa04916 Melanogenesis	0.01	hsa-let-7b-5p; hsa-miR-132-3p; hsa-miR-141-3p; hsa-miR-200a-3p; hsa-miR-200b-3p	0.02	hsa-miR-199a-5p; hsa-miR-27b-3p; hsa-miR-374a-5p; hsa-miR-374b-5p
hsa05014 Amyotrophic lateral sclerosis ALS	0.05	hsa-miR-141-3p; hsa-miR-200a-3p; hsa-miR-200b-3p; hsa-miR-296-5p		
hsa05212 Pancreatic cancer	0.02	hsa-let-7b-5p; hsa-miR-132-3p; hsa-miR-141-3p; hsa-miR-200a-3p; hsa-miR-200b-3p; hsa-miR-296-5p		
hsa05213 Endometrial cancer	0.04	hsa-let-7b-5p; hsa-miR-141-3p; hsa-miR-200a-3p; hsa-miR-200b-3p; hsa-miR-296-5p		

hsa05214 Glioma	0.03	hsa-let-7b-5p; hsa-miR-132-3p; hsa-miR-141-3p; hsa-miR-200a-3p; hsa-miR-200b-3p; hsa-miR-296-5p		
hsa05215 Prostate cancer	0.05	hsa-let-7b-5p; hsa-miR-132-3p; hsa-miR-141-3p; hsa-miR-200a-3p; hsa-miR-200b-3p; hsa-miR-296-5p		
hsa05216 Thyroid cancer	0.01	hsa-let-7b-5p; hsa-miR-141-3p; hsa-miR-200a-3p; hsa-miR-200b-3p; hsa-miR-296-5p		
hsa05217 Basal cell carcinoma	0.02	hsa-miR-132-3p; hsa-miR-141-3p; hsa-miR-200a-3p; hsa-miR-200b-3p		
hsa05220 Chronic myeloid leukemia	0.03	hsa-let-7b-5p; hsa-miR-132-3p; hsa-miR-141-3p; hsa-miR-200a-3p; hsa-miR-200b-3p; hsa-miR-296-5p		
hsa05221 Acute myeloid leukemia	0.02	hsa-let-7b-5p; hsa-miR-141-3p; hsa-miR-200a-3p; hsa-miR-200b-3p; hsa-miR-296-5p		
hsa05222 Small cell lung cancer	0.02	hsa-let-7b-5p; hsa-miR-132-3p; hsa-miR-141-3p; hsa-miR-200a-3p; hsa-miR-200b-3p; hsa-miR-296-5p		
hsa05412 Arrhythmogenic right ventricular cardiomyopathy ARVC	0.04	hsa-let-7b-5p; hsa-miR-141-3p; hsa-miR-200a-3p; hsa-miR-200b-3p		
hsa00140 Steroid hormone biosynthesis			0.03	hsa-miR-199a-5p; hsa-miR-27b-3p
hsa00512 O Glycan biosynthesis			0.04	hsa-miR-27b-3p; hsa-miR-374b-5p
hsa04630 Jak STAT signaling pathway			0.04	hsa-miR-199a-5p; hsa-miR-27b-3p; hsa-miR-374a-5p; hsa-miR-374b-5p

Table S5. Experimentally validated interactions between DE-miRNAs in serum of patients who later developed liver failure and the target genes

**miRNA**	**Target**	**Number of evidences**	**Number of publications**	**evidence**	**hsa04115 p53 signaling pathway**	**hsa04630 Jak STAT signaling pathway**	**hsa04620 Toll like receptor signaling pathway**	**hsa04350 TGF beta signaling pathway**	**hsa00140 Steroid hormone biosynthesis**
hsa-let-7b-3p	CASP3	4	1	Immunohistochemistry//Luciferase reporter assay//qRT-PCR//Western blot	O				
hsa-let-7b-5p	CCND1	10	3	Immunoblot//Immunofluorescence//Luciferase reporter assay//qRT-PCR//Reporter assay;Other//immunoblot//Northern blot//Western blot//Microarray//Annexin V-FITC	O				
hsa-let-7b-5p	CCND2	4	2	Luciferase reporter assay//Immunohistochemistry//QRTPCR//Western blot	O				
hsa-let-7b-5p	CDK6	3	1	Luciferase reporter assay//Microarray//Western blot	O				
hsa-let-7b-5p	TNFRSF10B	2	1	qRT-PCR//Western blot	O				
hsa-let-7b-5p	ACVR1	3	1	Luciferase reporter assay//Microarray//qRT-PCR				O	
hsa-let-7b-5p	AKT2	3	1	Luciferase reporter assay//qRT-PCR//Western blot		O			
hsa-let-7b-5p	HRAS	3	1	Immunoblot//qRT-PCR//Western blot		O			
hsa-let-7b-5p	IFNB1	3	1	ELISA//Luciferase reporter assay//qRT-PCR		O			
hsa-let-7b-5p	PDGFRA	3	1	Luciferase reporter assay//Microarray//qRT-PCR		O			
hsa-let-7b-5p	TGFBR1	3	1	GFP reporter assay//Western blot//ELISA				O	
hsa-let-7b-5p	TLR4	1	1	Luciferase reporter assay			O		
hsa-miR-132-3p	CCNB1	1	1	Western blot;qRT-PCR	O				
hsa-miR-132-3p	CDKN1A	3	1	qRT-PCR//Luciferase reporter assay//Western blot	O				
hsa-miR-132-3p	EGFR	4	1	Immunohistochemistry//Luciferase reporter assay//qRT-PCR//Western blot		O			
hsa-miR-132-3p	GDF5	2	1	Luciferase reporter assay//Western blot				O	
hsa-miR-132-3p	IRAK4	1	1	Luciferase reporter assay			O		
hsa-miR-132-3p	MAPK1	4	1	Luciferase reporter assay//QRTPCR//Microarray//Western blot			O		

hsa-miR-132-3p	PIK3R3	3	1	Luciferase reporter assay//qRT-PCR//Western blot		O			
hsa-miR-132-3p	RAF1	4	1	Immunohistochemistry//Luciferase reporter assay//qRT-PCR//Western blot		O			
hsa-miR-132-3p	SMAD2	3	1	GFP reporter assay//qRT-PCR//Western blot				O	
hsa-miR-133a-3p	BCL2L1	4	2	Luciferase reporter assay//Western blot//ChIP-seq//qRT-PCR	O				
hsa-miR-133a-3p	CASP9	1	1	Luciferase reporter assay	O				
hsa-miR-133a-3p	IGF1	1	1	Luciferase reporter assay	O				
hsa-miR-133a-3p	EGFR	4	5	Luciferase reporter assay//Western blot//qRT-PCR//Flow		O			
hsa-miR-133a-3p	MCL1	4	2	Luciferase reporter assay//Western blot//ChIP-seq//qRT-PCR		O			
hsa-miR-133a-3p	PIK3R2	1	1	Reporter assay		O			
hsa-miR-133a-3p	SP1	3	2	Luciferase reporter assay//Immunofluorescence//Western blot				O	
hsa-miR-141-3p	PTEN	3	1	Luciferase reporter assay//Western blot//Reporter assay;Western blot;Other	O				
hsa-miR-141-3p	ACVR2B	2	1	Luciferase reporter assay//Western blot				O	
hsa-miR-141-3p	MAPK14	3	1	Luciferase reporter assay//Microarray//Western blot			O		
hsa-miR-141-3p	MAPK9	1	1	Western blot			O		
hsa-miR-141-3p	STAT4	4	1	Luciferase reporter assay//qRT-PCR//Western blot//Immunohistochemistry		O			
hsa-miR-141-3p	STAT5A	3	1	Immunocytochemistry//Western blot//Luciferase reporter assay		O			
hsa-miR-141-3p	TGFB2	5	2	Immunoblot//Luciferase reporter assay//qRT-PCR//Flow//Western blot				O	
hsa-miR-199a-5p	ACVR1B	4	1	Immunoprecipitaion//qRT-PCR//Western blot//Luciferase reporter assay				O	
hsa-miR-199a-5p	IKBKB	5	1	Reporter assay//Western blot//GFP reporter assay//Luciferase reporter assay//qRT-PCR			O		
hsa-miR-199a-5p	LIF	2	1	ELISA//Luciferase reporter assay		O			
hsa-miR-199a-5p	NFKB1	3	1	Luciferase reporter assay//qRT-PCR//Western blot			O		
hsa-miR-199a-5p	PIAS3	3	1	Luciferase reporter assay//qRT-PCR//Western blot		O			
hsa-miR-199a-5p	PIK3CD	2	1	Luciferase reporter assay//qRT-PCR		O			

hsa-miR-199a-5p	SMAD3	1	1	Luciferase reporter assay				O	
hsa-miR-199a-5p	SMAD4	1	2	Luciferase reporter assay				O	
hsa-miR-199a-5p	SULT1E1	1	1	Luciferase reporter assay					O
hsa-miR-199a-5p	TGFB2	2	1	Luciferase reporter assay//qRT-PCR				O	
hsa-miR-199a-5p	TGFBR1	4	1	Luciferase reporter assay//Microarray//qRT-PCR//Western blot				O	
hsa-miR-200a-3p	CCNE2	1	1	Luciferase reporter assay	O				
hsa-miR-200a-3p	CDK6	3	1	Luciferase reporter assay//qRT-PCR//Western blot	O				
hsa-miR-200a-3p	PTEN	2	1	qRT-PCR//Western blot	O				
hsa-miR-200a-3p	TP53	2	1	Luciferase reporter assay//Western blot	O				
hsa-miR-200a-3p	EGFR	1	1	Western blot		O			
hsa-miR-200a-3p	GRB2	1	1	Luciferase reporter assay		O			
hsa-miR-200a-3p	MAPK14	3	1	Luciferase reporter assay//Microarray//Western blot			O		
hsa-miR-200a-3p	SMAD2	1	1	Luciferase reporter assay				O	
hsa-miR-200a-3p	SMAD3	1	1	Luciferase reporter assay				O	
hsa-miR-200a-3p	TGFB2	3	1	Luciferase reporter assay//qRT-PCR//Western blot				O	
hsa-miR-200b-3p	BCL2	3	1	Luciferase reporter assay//qRT-PCR//Western blot	O				
hsa-miR-200b-3p	CCNE2	1	1	Luciferase reporter assay	O				
hsa-miR-200b-3p	IKBKB	3	1	Luciferase reporter assay//qRT-PCR//Western blot			O		
hsa-miR-200b-3p	JUN	3	1	Luciferase reporter assay//qRT-PCR//Western blot			O		
hsa-miR-200b-3p	RHOA	1	1	Luciferase reporter assay				O	
hsa-miR-200b-3p	SMAD2	1	1	Luciferase reporter assay				O	
hsa-miR-200b-3p	SP1	4	2	In situ hybridization//Luciferase reporter assay//qRT-PCR//Western blot				O	

hsa-miR-27b-3p	CCNG1	5	1	Immunohistochemistry//In situ hybridization//Luciferase reporter assay//qRT-PCR//Western blot	O				
hsa-miR-27b-3p	THBS1	1	1	Luciferase reporter assay	O				
hsa-miR-27b-3p	CYP1B1	5	2	Immunohistochemistry//Luciferase reporter assay//qRT-PCR//Western blot//Reporter assay;Other					O
hsa-miR-27b-3p	CYP3A4	2	1	Immunoblot//Luciferase reporter assay					O
hsa-miR-27b-3p	EGFR	3	1	qRT-PCR//Luciferase reporter assay//Western blot		O			
hsa-miR-27b-3p	SMAD2	2	1	Luciferase reporter assay//Western blot				O	
hsa-miR-27b-3p	TGFBR1	2	1	Luciferase reporter assay//Western blot				O	
hsa-miR-296-5p	BBC3	3	1	Luciferase reporter assay//qRT-PCR//Western blot	O				
hsa-miR-296-5p	CASP8	3	1	Immunoprecipitaion//Luciferase reporter assay//qRT-PCR	O				
hsa-miR-296-5p	IKBKE	1	1	Luciferase reporter assay			O		
hsa-miR-374a-5p	ATM	3	1	Immunoblot//Luciferase reporter assay//qRT-PCR	O				
hsa-miR-374a-5p	CCND1	2	1	Luciferase reporter assay//Western blot	O				
hsa-miR-374a-5p	GADD45 A	3	1	Immunoblot//Luciferase reporter assay//qRT-PCR	O				
hsa-miR-374b-5p	AKT1	4	1	Luciferase reporter assay//Microarray//qRT-PCR//Western blot		O			
hsa-miR-376a-3p	CASP8	3	1	Luciferase reporter assay//qRT-PCR//Western blot	O				
hsa-miR-376a-3p	CDK2	1	1	Luciferase reporter assay	O				
hsa-miR-376a-3p	ACVR1C	2	1	Luciferase reporter assay//Reporter assay;Western blot				O	
hsa-miR-376a-3p	PIK3R1	3	1	Luciferase reporter assay//qRT-PCR//Western blot		O			
hsa-miR-376c-3p	BCL2	4	1	Flow//Immunohistochemistry//qRT-PCR//Western blot	O				
hsa-miR-376c-3p	ACVR1C	4	2	Luciferase reporter assay//Western blot//Reporter assay;Western blot;qRT-PCR//qRT-PCR				O	
hsa-miR-376c-3p	GRB2	3	1	Luciferase reporter assay//qRT-PCR//Western blot		O			
hsa-miR-376c-3p	TGFBR1	3	1	Luciferase reporter assay//qRT-PCR//Western blot				O	
hsa-miR-376c-3p	UGT2B1 5	2	2	Luciferase reporter assay//qRT-PCR					O

hsa-miR-376c-3p	UGT2B17	2	2	Luciferase reporter assay//qRT-PCR					O
hsa-miR-584-5p	ROCK1	5	1	Immunohistochemistry//Luciferase reporter assay//Microarray//qRT-PCR//Western blot				O	
hsa-miR-7-5p	BAX	1	1	Luciferase reporter assay	O				
hsa-miR-7-5p	BCL2	5	2	GFP reporter assay//Luciferase reporter assay//qRT-PCR//Western blot//Reporter assay;Western blot;qRT-PCR;Other	O				
hsa-miR-7-5p	CCNE1	4	2	Flow//Luciferase reporter assay//qRT-PCR//Western blot	O				
hsa-miR-7-5p	SERPINB5	7	2	Immunoprecipitaion//Luciferase reporter assay//Microarray//qRT-PCR//Western blot//ChIP-seq//Immunohistochemistry	O				
hsa-miR-7-5p	EGFR	11	12	real-time RT-PCR//Reporter assay//Western blot//Luciferase reporter assay//Reporter assay;Western blot;qRT-PCR;Other//qRT-PCR//Reporter assay;Western blot;qRT-PCR;Microarray;Other//Microarray//Immunohistochemistry//In situ hybridization//Flow		O			
hsa-miR-7-5p	FOS	8	1	ChIP-seq//Immunofluorescence//Immunohistochemistry//In situ hybridization//Luciferase reporter assay//Northern blot//qRT-PCR//Western blot			O		
hsa-miR-7-5p	GDF5	3	1	Luciferase reporter assay//qRT-PCR//Western blot				O	
hsa-miR-7-5p	PIK3CD	3	2	Luciferase reporter assay//qRT-PCR//Western blot		O			
hsa-miR-7-5p	PIK3R3	4	1	ELISA//Luciferase reporter assay//qRT-PCR//Western blot		O			
hsa-miR-7-5p	RAF1	5	2	Luciferase reporter assay//Microarray//qRT-PCR//Western blot//Reporter assay;qRT-PCR;Microarray;Other		O			
hsa-miR-7-5p	RELA	8	3	qRT-PCR//Western blot//ChIP-seq//Immunofluorescence//Immunohistochemistry//In situ hybridization//Luciferase reporter assay//Northern blot			O		
