# Supplementary material for: Serum miRNA profiles are altered in patients with primary sclerosing cholangitis receiving high-dose ursodeoxycholic acid
Source: JHEP Rep. 2023 Mar 23;5(6):100729. doi: 10.1016/j.jhepr.2023.100729 (PMC10172698; doi:10.1016/j.jhepr.2023.100729)
Supplement: Multimedia component 2 [file mmc2.docx]

**Journal of Hepatology**

**CTAT methods**

Tables for a “Complete, Transparent, Accurate and Timely account” (CTAT) are now mandatory for all revised submissions. The aim is to enhance the reproducibility of methods.

- Only include the parts relevant to your study
- Refer to the CTAT in the main text as ‘Supplementary CTAT Table’
- Do not add subheadings
- Add as many rows as needed to include all information
- Only include one item per row

**If the CTAT form is not relevant to your study, please outline the reasons why:**

|  |
| --- |

- 1. **Antibodies**

| **Name** | **Citation** | **Supplier** | **Cat no.** | **Clone no.** |
| --- | --- | --- | --- | --- |
| **N.A** | **N/A** | **N/A** | **N/A** | **N/A** |

- 1. **Cell lines**

| **Name** | **Citation** | **Supplier** | **Cat no.** | **Passage no.** | **Authentication test method** |
| --- | --- | --- | --- | --- | --- |
| **N/A** | **N/A** | **N/A** | **N/A** | **N/A** | **N/A** |

- 1. **Organisms**

| **Name** | **Citation** | **Supplier** | **Strain** | **Sex** | **Age** | **Overall n number** |
| --- | --- | --- | --- | --- | --- | --- |
| **N/A** | **N/A** | **N/A** | **N/A** | **N/A** | **N/A** | **N/A** |

- 1. **Sequence based reagents**

| **Name** | **Sequence** | **Supplier** |
| --- | --- | --- |
| **See below** | **N/A** | **N/A** |

- 1. **Biological samples**

| **Description** | **Source** | **Identifier** |
| --- | --- | --- |
| **Serum Samples** | **Prior study** “Lindor KD, Kowdley KV, Luketic VA, Harrison ME, McCashland T, Befeler AS, Harnois D, et al. High-dose ursodeoxycholic acid for the treatment of primary sclerosing cholangitis. Hepatology 2009;50:808-814.” | **N/A** |

- 1. **Deposited data**

| **Name of repository** | **Identifier** | **Link** |
| --- | --- | --- |
|  |  |  |

- 1. **Software**

| **Software name** | **Manufacturer** | **Version** |
| --- | --- | --- |
| **N/A** | **N/A** | **N/A** |

- 1. **Procedures used**

| **RNA isolation** | As mentioned in the study “ **Li** Y, Kowdley KV. Method for Microrna Isolation from Clinical Serum Samples. Analytical biochemistry 2012” | **N/A** |
| --- | --- | --- |
| **RNA extraction** | Synthetic-CEL-Mir 54 | **N/A** |
| **miRNA real time PCR** |  | **N/A** |
| Quantification of spike-in-control | Taqman miRNA real-time qPCR | **N/A** |
| Probes used for quantification | Taqman cel-miR-54 probe and Taqman Universal Master Mix No AmpErase^®^ UNG | **N/A** |
| PCR instrument | ABI-7500 real-time PCR instrument | **N/A** |
| **miRNA profiling** | miRCURY LNA^TM^ miRNA real-time PCR Human Panel I (Exiqon, Denmark | **N/A** |
| cDNA synthesis | miRCURY Locked Nucleic Acid (LNA) universal cDNA synthesis kit (Exiqon, Denmark) | **N/A** |
| Real-time qPCR | SYBR Green Master Mix (Exiqon, Denmark) on an ABI-7900HT real-time PCR instrument (Life Technologies, CA) | **N/A** |
| **Cytokine analysis** |  | **N/A** |
| Analysis of 27 cytokines | Bio-Plex^®^ Precision Pro™ Human Cytokine Assays (Bio-Rad, CA) on a Luminex 100 instrument (Luminex, TX), | **N/A** |
| **Imputation** | Impute R/Bioconductor package using the kNN imputation algorithm | **N/A** |
| **Identification of differentially expressed miRNA’s** | LIMMA R/Bioconductor package | **N/A** |
| **Biological process/pathway enrichment analysis** | miEAA software | **N/A** |
| **k-TOP scoring pairs classifier** | SwitchboxR/Bioconductor package to identify a set of paired miRNA classifiers. | **N/A** |
|  |  |  |

- 1. **Please provide the details of the corresponding methods author for the manuscript:**

| Kris Kowdley,  Director, Liver Institute Northwest  Professor  Elson S. Floyd College of Medicine  Washington State University  3216 NE 45^th^ Place, Suite 212  Seattle, WA 98105, USA |
| --- |

**2.0 Please confirm for randomised controlled trials all versions of the clinical protocol are included in the submission. These will be published online as supplementary information.**

| NIH grants 3R01DK056924-08S1, 5K24DK002957 and 1R21HL112678 |
| --- |
