# Supplementary material for: Serum miRNA profiles are altered in patients with primary sclerosing cholangitis receiving high-dose ursodeoxycholic acid
Source: JHEP Rep. 2023 Mar 23;5(6):100729. doi: 10.1016/j.jhepr.2023.100729 (PMC10172698; doi:10.1016/j.jhepr.2023.100729)
Supplement: Multimedia component 4 [file mmc4.pdf]

Research article

JHEP Reports

Serum miRNA profiles are altered in patients with primary sclerosing cholangitis receiving high-dose ursodeoxycholic acid

Authors

Jessica T. Hochberg, Aalam Sohal, Priya Handa, Bryan D. Maliken, Take-Kyun Kim, Kai Wang, Eric Gochanour, Yu Li, J. Bart Rose, James E. Nelson, Keith D. Lindor, Nicholas F. LaRusso, Kris V. Kowdley

Correspondence

kkowdley@liverinstitutenw.org (K.V. Kowdley), jessica.hochberg@seattlechildrens.org, Jessica.hochberg@gmail.com (J.T. Hochberg).

Graphical abstract

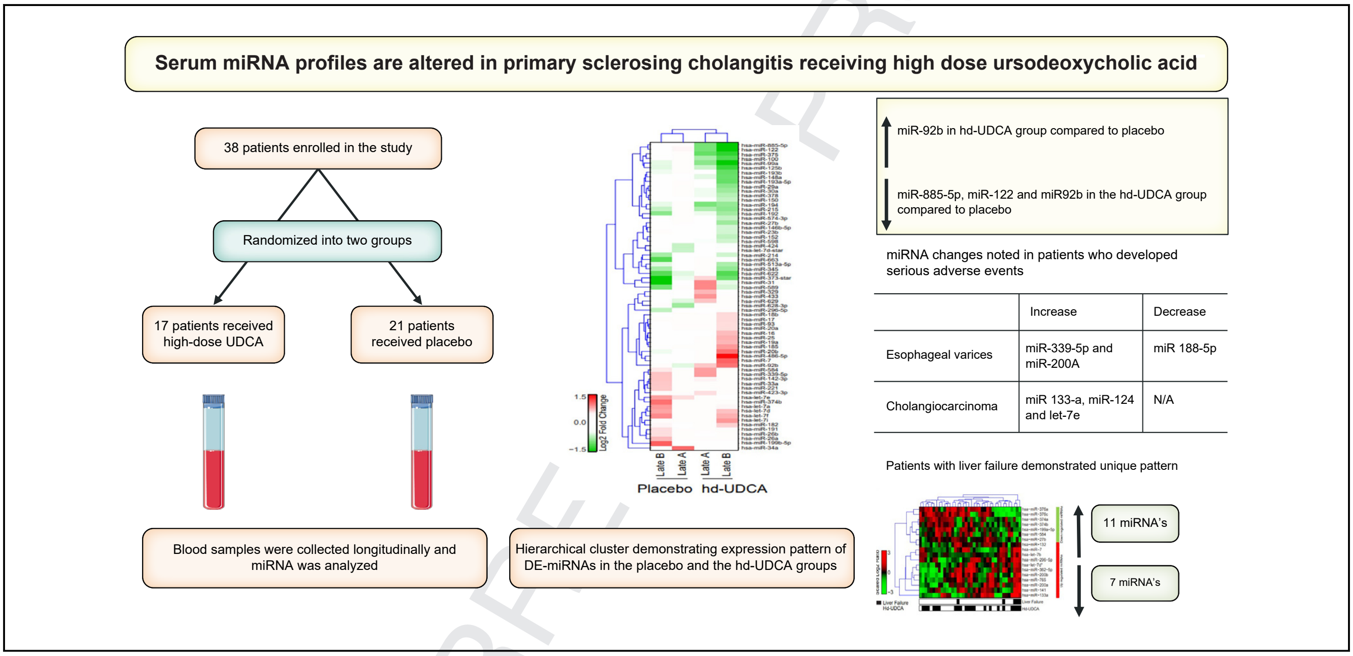

Highlights

- Longitudinal changes occurred in miRNA profiles of patients treated with hd-UDCA.
- The concentration of miR-92b, an oncogene, increased in patients treated with hd-UDCA.
- The concentration of miR-122, miR-885-5p, miR-375, and miR-99a decreased in patients treated with hd-UDCA.
- Patients who progressed to liver failure resulting in liver-related death or liver transplantation demonstrated a unique miRNA pattern with 18 DE-miRNAs.

Impact and implications

Using serum samples from patients with PSC enrolled in a clinical trial comparing hd-UDCA with placebo, our study found distinct miRNA changes in patients with PSC who are treated with hd-UDCA over a period of time. Our study also noted distinct miRNA patterns in patients who developed SAEs during the study period.

UNCORRECTED PROOF

https://doi.org/10.1016/j.jhepr.2023.100729

UNCORRECTED PROOF

Serum miRNA profiles are altered in patients with primary sclerosing cholangitis receiving high-dose ursodeoxycholic acid

Jessica T. Hochberg,[1,2,3] Aalam Sohal,[1] Priya Handa,[4] Bryan D. Maliken,[4] Take-Kyun Kim,[5] Kai Wang,[5] Eric Gochanour,[1] Yu Li,[4] J. Bart Rose,[4] James E. Nelson,[4] Keith D. Lindor,[6] Nicholas F. LaRusso,[6] Kris V. Kowdley[1,*]

[1]Liver Institute Northwest, Seattle, WA, USA; [2]Seattle Children's Hospital/University of Washington, Seattle, WA, USA; [3]Miami Transplant Institute at University of Miami, Miami, FL, USA; [4]Benaroya Research Institute, Seattle, WA, USA; [5]Institute for Systems Biology, Seattle, WA, USA; [6]Division of Gastroenterology and Hepatology, Mayo Clinic Rochester, MN, USA

JHEP Reports **2023.** https://doi.org/10.1016/j.jhepr.2023.100729

Background & Aims: Primary sclerosing cholangitis (PSC) is a chronic, progressive cholestatic liver disease that can lead to end-stage liver disease and cholangiocarcinoma. High-dose ursodeoxycholic acid (hd-UDCA, 28–30 mg/kg/day) was evaluated in a previous multicentre, randomised placebo-controlled trial; however, the study was discontinued early because of increased liver-related serious adverse events (SAEs), despite improvement in serum liver biochemical tests. We investigated longitudinal changes in serum miRNA and cytokine profiles over time among patients treated with either hd-UDCA or placebo in this trial as potential biomarkers for PSC and response to hd-UDCA, as well as to understand the toxicity associated with hd-UDCA treatment.
Methods: Thirty-eight patients with PSC were enrolled in a multicentred, randomised, double-blinded trial of hd-UDCA *vs.* placebo.
Results: Significant alterations in serum miRNA profiles were found over time in both patients treated with hd-UDCA or placebo. Additionally, there were striking differences between miRNA profiles in patients treated with hd-UDCA compared with placebo. In patients treated with placebo, the changes in concentration of serum miRNAs miR-26a, miR-199b-5p, miR-373, and miR-663 suggest alterations of inflammatory and cell proliferative processes consistent with disease progression. However, patients treated with hd-UDCA exhibited a more pronounced differential expression of serum miRNAs, suggesting that hd-UDCA induces significant cellular miRNA changes and tissue injury. Pathway enrichment analysis for UDCA-associated miRNAs suggested unique dysregulation of cell cycle and inflammatory response pathways.
Conclusions: Patients with PSC have distinct miRNAs in the serum and bile, although the implications of these unique patterns have not been studied longitudinally or in relation to adverse events related to hd-UDCA. Our study demonstrates marked changes in miRNA serum profiles with hd-UDCA treatment and suggests mechanisms for the increased liver toxicity with therapy.
Impact and implications: Using serum samples from patients with PSC enrolled in a clinical trial comparing hd-UDCA with placebo, our study found distinct miRNA changes in patients with PSC who are treated with hd-UDCA over a period of time. Our study also noted distinct miRNA patterns in patients who developed SAEs during the study period.
© 2023 The Author(s). Published by Elsevier B.V. on behalf of European Association for the Study of the Liver (EASL). This is an open access article under the CC BY license (http://creativecommons.org/licenses/by/4.0/).

Introduction

Primary sclerosing cholangitis (PSC) is a progressive cholestatic liver disease of unknown etiopathogenesis, which can lead to end-stage liver disease, portal hypertension, and cholangiocarcinoma (CC), with a median survival of 15–20 years following diagnosis.[1] There are no approved therapies for PSC, with liver transplant being the only definitive therapy currently.

Ursodeoxycholic acid (UDCA), a secondary bile acid, has been used to treat cholestatic liver diseases. UDCA (13–15 mg/kg/day) is approved by the FDA for the treatment of primary biliary cholangitis, but not primary sclerosing cholangitis. The efficacy of high-dose UDCA (hd-UDCA, 28–30 mg/kg/day) as treatment for PSC was evaluated in a multicentre randomised placebo-controlled trial.[2] Although patients treated with hd-UDCA demonstrated improvement in serum liver biochemical tests, the trial was prematurely terminated because of an unexpected higher rate of liver-related serious adverse events (SAEs), including liver-related death, liver failure, and liver transplantation, among patients treated with hd-UDCA. The possible pathophysiological mechanisms for increased liver-related SAEs in patients treated with hd-UDCA have not been elucidated.

miRNAs are small non-coding RNAs that negatively regulate genes by promoting mRNA degradation and/or blocking translational initiation. Recent studies demonstrated the involvement

Keywords: Serum; miRNA; Biomarker; PSC; UDCA; High-dose.
Received 8 July 2022; received in revised form 2 February 2023; accepted 27 February 2023; available online 23 March 2023
* Corresponding author. Address: Liver Institute Northwest, 3216 NE 45th Pl Suite 212, Seattle, WA 98105, USA; Tel.: +1(206) 536-3030
E-mail address: kkowdley@liverinstitutenw.org (K.V. Kowdley).

of miRNAs in key liver functions, including the homeostasis of glucose,[3] cholesterol,[4] and iron.[5] Increasingly, miRNAs have been associated with numerous diseases, such as metabolic disorders, cancers, and various liver diseases, including cholangitis,[6] non-alcoholic fatty liver disease (NAFLD),[7] HCV,[8] HBV,[9] and hepatocellular carcinoma (HCC).[10] Cellular miRNAs are also released into the circulation, and may represent a new type of biomarker for PSC and other cholestatic liver diseases because they are: (i) protected from the high activity of RNase activities present in the extracellular environment by forming protein complexes or encapsulated in lipid vesicles;[11] (ii) stable for multiple freeze thaw cycles;[12] and (iii) readily measurable with common laboratory technologies, including next-generation sequencing (NGS) or quantitative reverse transcription (qRT)-PCR.[13] Most recently, extracellular vesicles containing miRNA were identified with the hopes of opening new opportunities for non-invasive diagnostic tools for patients with PSC.[14] Therefore, characterising miRNA profiles may provide novel insights into pathophysiology and mechanisms for liver injury and disease.

The goal of this study was to examine whether there were differences over time in serum miRNA profiles between patients treated with either placebo or hd-UDCA. Our hypothesis was that hd-UDCA therapy in PSC is associated with distinct changes in miRNA profiles compared with placebo.

Materials and methods

Patients

Patients were enrolled in a multicentered, randomised, double-blinded trial of hd-UDCA (28–30 mg/kg/day) versus placebo; details of this study have been previously published.[2] Institutional review board at each site approved the study. Thirty-eight subjects from the original cohort (placebo = 17, hd-UDCA = 21) with available serum samples were included in the present study. Blood samples from each patient from both baseline entry point and a second time point were included. The current study was approved by the institutional review board at Benaroya Research Institute, Seattle, WA and Mayo Clinic, Rochester, MN.

Serum samples

Blood samples were collected and processed as described previously.[15] Serum samples were stored in a −80 °C freezer before RNA extraction. We then examined the levels of 375 human miRNAs and 27 cytokines in serum samples from all 38 patients. We identified serum miRNA signatures associated with PSC progression, hd-UDCA treatment, and SAEs, including esophageal varices (EVs), CC, and liver failure.

RNA extraction

The protocol for RNA extraction from 200-μL serum was described previously.[15] Synthetic cel-miR-54 (20 *f*mol) (IDT DNA Technologies, IA, USA) was spiked into each serum sample during the RNA extraction process for normalisation. Total RNA extracted from 200-μL serum was eluted from the RNA isolation column in 50-μL ddH_2O.

TaqMan miRNA real-time qPCR

TaqMan miRNA real-time qPCR was performed to quantify the cel-miR-54 spike-in control. Briefly, 5 μL of total RNA was reversely transcribed in a 15-μL reaction volume for each sample using the TaqMan miRNA reverse-transcription kit (Thermo Fisher Scientific, San Francisco, CA, USA) according to the manufacturer's protocol. TaqMan cel-miR-54 probe and TaqMan Universal Master Mix No AmpErase® UNG were purchased from Thermo Fisher Scientific (Thermo Fisher Scientific). Real-time qPCR was performed on an ABI-7500 real-time PCR instrument (Life Technologies) according to the manufacturer's protocol.

miRNA profiling

miRNA profiling was performed using the miRCURY LNA™ miRNA real-time PCR Human Panel I (Exiqon, Copenhagen, Denmark). The panel was designed based on the miRBase release version 14 and contained probes for 375 human miRNAs. For miRNA profiling, 15 μL of total RNA was reversely transcribed in a 40-μL reaction volume for each sample. The miRCURY Locked Nucleic Acid (LNA) universal cDNA synthesis kit (Exiqon) was used to make cDNA for miRNA profiling according to the manufacturer's protocol. Real-time qPCR was performed using the SYBR Green Master Mix (Exiqon) on an ABI-7900HT real-time PCR instrument (Life Technologies) according to the manufacturer's protocol.

Cytokine analysis

Twenty-seven cytokines levels were measured using Bio-Plex® Precision Pro™ Human Cytokine Assays (Bio-Rad Laboratories, Hercules, CA, USA) on a Luminex 100 instrument (Luminex, Austin, TX, USA), according to the manufacturers' protocols. Twenty-seven cytokines were analysed: IL-1β, IL-1ra, IL-6, IL-7, IL-8, IL-13, IL-15, IL-17, IL-18, fibroblast growth factor (FGF)-basic, eotaxin, granulocyte colony-stimulating factor (G-CSF), GRO-α, hepatocyte growth factor (HGF), interferon (IFN)-γ, interferon gamma-induced protein 10 (IP-10), leukemia inhibitory factor (LIF), macrophage migration inhibitory factor (MIF), monocyte chemoattractant protein 1 (MCP-1), macrophage inflammatory protein (MIP)-1α, MIP-1β, platelet-derived growth factor (PDGF)-BB, regulated upon activation, normal T cell expressed and presumably secreted (RANTES), stem cell factor (SCF), tumour necrosis factor (TNF)-α, vascular endothelial growth factor (VEGF), and tumour necrosis factor-related apoptosis-inducing ligand (TRAIL).

Data analysis

A data matrix comprising cycle threshold (Ct) values for miRNAs and 104 samples was prepared. Average values for miRNAs with Ct <35 were computed at each sample. Mean normalisation was then performed by subtracting the average value from individual miRNA Ct values at each sample. miRNAs with more than 20% missing values across the samples were removed. The remaining missing values were imputed with the impute R/Bioconductor package using the kNN imputation algorithm.[16] Data quality assessment was then performed with either a scatter plot or Pearson correlation coefficient between samples.

miRNAs differentially expressed between the two groups were identified by LIMMA R/Bioconductor package[17] with the criteria of p <0.05 and log2-fold-change >0.585 for upregulation or <−0.585 for downregulation. Biological process/pathway enrichment analysis was performed with miEAA software.[18] Changes in serum cytokine concentration between the two groups were assessed using the Mann-Whitney U test. A p <0.05 was considered statistically significant. k-TOP scoring pairs classifier was performed with switchbox R/Bioconductor package[19] to identify a set of paired miRNA classifiers.

Table 1. Characteristics of patients included in the study.

Patient characteristics	Placebo (n = 17)	hd-UDCA (n = 21)	*p* value
Age (years)	42.0 (33.5–48.5)	44.0 (37–49)	0.433
Sex (male, %)	68.8	54.5	0.506
Blood chemistry*			
Aspartate aminotransferase	2.7 (1.6–3.7)	2.6 (1.7–4.0)	0.830
Alkaline phosphatase	3.2 (2.3–4.9)	3.8 (2.7–4.8)	0.702
Total bilirubin	1.0 (0.6–1.2)	0.8 (0.7–1.0)	0.559
Albumin	1.2 (1.1–1.3)	1.2 (1.0–1.2)	0.739

Data are presented as median (36) unless otherwise indicated.
* All laboratory values are reported as multiples of upper limits of normal.

Table 3. Number of patients in the hd-UDCA and placebo groups who progressed to the primary endpoint.

Primary endpoint	Placebo (n = 17), n (%)	hd-UDCA (n = 21), n (%)
End cirrhosis	1 (4.5)	1 (6.3)
End esophageal varices	5 (22.7)	6 (37.5)
Cholangiocarcinoma	2 (9.1)	2 (12.5)
Transplant	0	2 (12.5)
Death	0	4 (25)

Results

Patient characteristics

There were no significant differences in age, sex, and serum levels of aspartate transaminase (AST), alkaline phosphatase (ALP), and total bilirubin at entry between patients in the hd-UDCA group and the placebo group (Table 1). Patients in the hd-UDCA group showed decreased levels of AST and ALP at post-entry points compared with both their entry time point and matched time points of the placebo group. The placebo group showed no difference in AST, ALP, and total bilirubin when comparing entry to all later time points (Table 2). However, the hd-UDCA group exhibited more SAEs, such as liver failure resulting in liver transplantation (0% *vs.* 9.1%) and liver-related death (0% *vs.* 18.2%), compared with the placebo group (Table 3).

The placebo group and the hd-UDCA group exhibited distinct time-associated serum miRNA patterns

We compared the post-entry serum miRNA profiles of patients receiving placebo or hd-UDCA with their profiles at entry to identify time-associated miRNA changes within each group. The post-entry profiles were assigned to one of two groups: Year 1–4 (Late A), and Year 5 (Late B). Both groups experienced an increase in the number of differentially expressed miRNAs (DE-miRNAs) over time, but the change was more pronounced in the hd-UDCA group. In the placebo group, the number of DE-miRNAs increased from four at Late A (Year 1–4) to 21 at Late B (Year 5) (Fig. 1A). By comparison, the number of DE-miRNAs in the hd-UDCA group increased from 16 in Late A to 42 in Late B (Fig. 1A). Overall, 65 DE-miRNAs showed significant concentration changes (fold change >1.5, p <0.05) in at least one post-entry time point in either the placebo or hd-UDCA group (Table S1). We used an unsupervised hierarchical cluster to illustrate the changes in these 65 miRNAs in both the placebo and hd-UDCA groups. All groups from two post-entry time points were clustered according to treatment, suggesting that the patients treated with placebo and those treated with hd-UDCA had distinct serum miRNA profiles (Fig. 1B). In contrast to miRNA, we did not observe significant time-associated concentration changes for the 27 cytokines measured in serum.

The changes in DE-miRNAs between the two time points within the patient groups were also examined. Only one miRNA, miR-92b, showed an increased concentration at both time points (Fig. 1C), while the levels of seven miRNAs (miR-100, miR-122, miR-125b, miR-194, miR-215, miR-375, and miR-99a) decreased at both time points within the hd-UDCA group. There was no common DE-miRNA between the two time points within the placebo group (Fig. 1D).

hd-UDCA treatment altered serum miRNA and cytokine profiles in patients with PSC

Next, we compared the post-entry serum miRNA profiles between patients treated with hd-UDCA and the placebo group to identify the impact of hd-UDCA treatment on the serum miRNA profile. Based on the same selection criteria used above, 43 DE-miRNAs were identified (Table S2), which grouped into five patterns (C1–C5; Fig. 2A). The C1 and C2 clusters were miRNAs affected only at the Late A time point, which comprised 20 miRNAs with increased concentrations and 10 miRNAs with decreased concentrations. The C3 and C4 clusters were miRNAs affected at the Late B time point, which comprised eight miRNAs with increased concentrations and four with decreased concentrations. The C5 cluster contained only one miRNA, miR-493, which showed an increased concentration between entry and Late A. Although there were no miRNAs showing statistically significant concentration changes between the two consecutive time points, some miRNAs showed the same trend in concentration change at both time points. For example, miR-31, miR-185*, miR-92b, and miR-887 showed a >1.5-fold change at both time points even though p >0.05 at Late B. Interestingly, miR-31 has been documented to be involved in the pathogenesis of liver fibrosis,[20] and it has been shown that miR-92b promotes HCC progression.[21] In addition, the concentrations of miR-122, miR-885-5p, miR-375, and miR-99a showed a >1.5-fold decrease at both time points. Notably, miR-122, a liver-enriched miRNA involved in liver homeostasis, demonstrated the greatest concentration decrease upon hd-UDCA treatment at Late A time-point (fold change = 0.43, p = 5.25E-03). Recently, the serum miR-

Table 2. Serum levels of aspartate transaminase, alanine transaminase, and total bilirubin between two groups.*

	n		Aspartate transaminase			Alanine transaminase			Total bilirubin		
Study time point	Placebo	hd-UDCA	Placebo	hd-UDCA	*p* value	Placebo	hd-UDCA	*p* value	Placebo	hd-UDCA	*p* value
Baseline (at entry)	22	16	2.7 (1.6–3.7)	2.6 (1.7–4.0)	0.830	3.2 (2.3–4.9)	3.8 (2.7–4.8)	0.702	1.0 (0.6–1.2)	0.8 (0.7–1.0)	0.559
12 months	21	16	2.7 (1.6–3.3)	1.1 (0.8–2.2)	**0.014**	1.9 (1.5–2.5)	1.9 (1.5–2.5)	**0.003**	1.0 (0.7–1.4)	0.7 (0.6–1.0)	0.115
24 months	22	15	2.4 (1.5–3.3)	1.4 (1.1–2.0)	0.055	1.9 (1.2–2.6)	1.9 (1.2–2.6)	**0.027**	0.9 (0.6–1.6)	0.9 (0.6–1.5)	0.710
36 months	17	15	2.5 (1.6–4.0)	1.3 (0.7–1.8)	**0.007**	1.6 (1–2.5)	1.6 (1.0–2.5)	**0.001**	0.9 (0.6–2.0)	1.0 (0.7–1.4)	0.584

Data are presented as the median (36) of upper limits of normal laboratory value. Bold suggests statistical significance.
* Mann-Whitney *U* test was used to compare the groups.

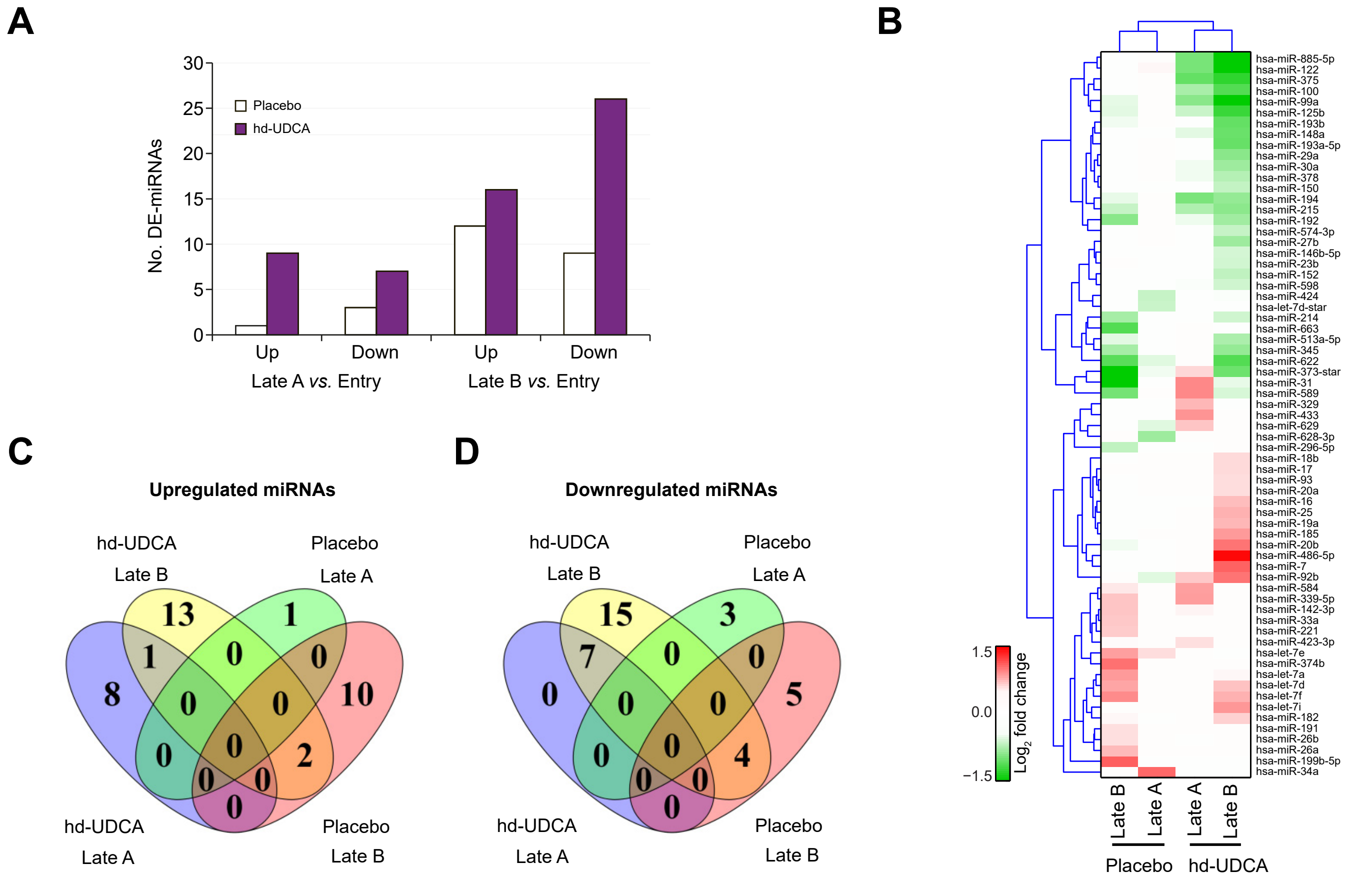

Fig. 1. Patients treated with hd-UDCA *vs.* placebo exhibit distinct serum cell-free miRNA patterns. The miRNA signatures with significant expression changes were selected using a cut-off of a fold change >1.5 and p <0.05. The changes in miRNA in patients receiving placebo or hd-UDCA in post-entry time points are relative to the corresponding entry time point. (A) Numbers of miRNA with significant changes in concentration at different post-entry time points. (B) Unsupervised hierarchical cluster demonstrating the expression pattern of DE-miRNAs in the placebo and hd-UDCA groups at different post-entry time points. Red represents increased concentrations; green represents decreased concentrations. A total of 67 DE-miRNAs passed the cut-off and were included in the analysis. (C). Relationships of DE-miRNAs at increased concentrations in hd-UDCA or placebo compared with baseline sample at entry. (D) Relationships of DE-miRNAs at decreased concentration in either hd-UDCA or placebo groups compared with sample at entry. DE, differentially expressed; hd-UDCA, high-dose ursodeoxycholic acid.

885-5p level was reported as a potential biomarker to detect liver pathologies, including HCC, liver cirrhosis, and chronic hepatitis B.[22]

To investigate the biological processes represented by DE-miRNAs observed in hd-UDCA, pathway enrichment analysis was performed for miRNAs in each cluster by using the miEAA tool (Table S3).[18] MiRNAs in clusters C1 and C3 were associated with ABC transporters and cell cycle-related pathways (*e.g.* DNA replication in C1 and cell cycle and DNA damage response in C3) (Fig. 2B). Clusters C2 and C4 showed decreased concentrations in miRNAs associated with adipogenesis, ATM-dependent DNA damage response, calcium signaling pathways, leptin signaling pathway, inositol phosphate metabolism, oxidative stress response, and the insulin–IGF pathway protein kinase B signaling cascade. Cluster C2 miRNAs were also associated with several immune/inflammation-related responses [*e.g.* antigen processing and presentation, complement and coagulation cascades, B/T cell activation, transforming growth factor (TGF)-β signaling pathways, etc.], which may represent the involvement of inflammation in PSC pathogenesis. Taken together, these data show that hd-UDCA treatment markedly impacted the serum miRNA profiles in patients with PSC and suggest that UDCA promoted pathways leading to liver-related adverse events.

We also compared the serum cytokine profiles between patients treated with hd-UDCA and the placebo group, and identified only one cytokine with a significant concentration difference between the two groups. The serum level of TRAIL decreased in both groups post-entry, but showed a greater reduction in the hd-UDCA group [mean = −76% (−84.2% to −61.9%)] compared with the placebo group [mean = −31.4% (−65.1% to −4.7%)], with p = 0.018 (Fig. 2C).

Serum miRNA profile may associate with SAEs

We also investigated whether there were specific circulating miRNAs associated with the SAEs observed in the study. Increased serum concentrations of miR-339-5p and miR-200a, and decreased levels of miR-188-5p, were observed in patients who developed EVs (Table 4), compared with patients without EV. Increased concentrations of miR-133a, miR-124, and let-7e were seen in patients who developed CC (Table 4), compared with patients without CC. Notably, patients who progressed to liver failure resulting in liver-related death or liver transplantation demonstrated a unique miRNA pattern with 18 DE-

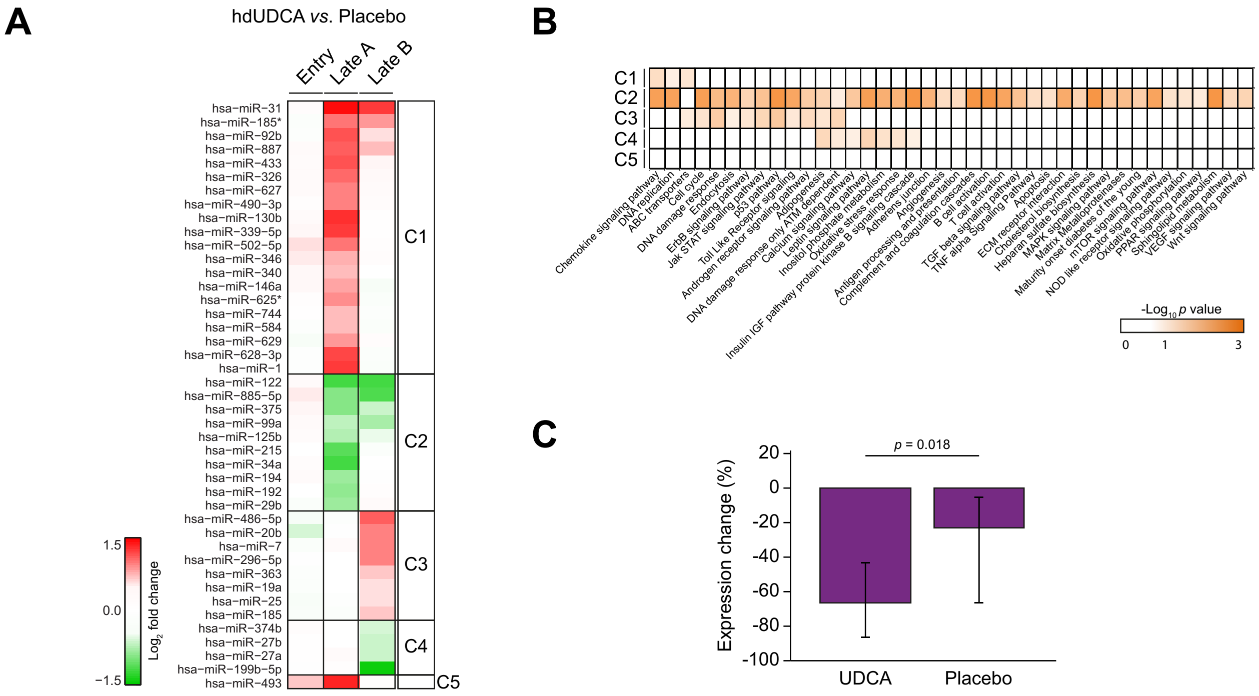

Fig. 2. hd-UDCA treatment alters the levels of a group of miRNAs and the cytokine TRAIL in serum. (A) Heat map of the changes in concentration of serum miRNA signatures in the hd-UDCA patient group compared with the placebo group at three different time points (entry, Late A, and Late B). The miRNAs can be grouped in five different clusters (C1–C5) based on the changes in concentration at different time points. (B) Heat map of the significance of pathways enriched by DE-miRNAs in each cluster. The scale indicates $-\log_{10}$ transformed p values, with the darker colour indicating greater significance. Level of significance: p <0.05, Fisher's exact test. (C) Serum concentration of the TRAIL cytokine between patients treated with hd-UDCA and those treated with placebo. The percentage change represents the expression level change at post-entry time points relative to the baseline level at entry. Data are presented as mean ± SD. Level of significance: p <0.05, Student's t test. DE, differentially expressed; hd-UDCA, high-dose ursodeoxycholic acid.

Table 4. Serum miRNAs associated with serious adverse events after hd-UDCA treatment.

Serious adverse event	miRNA	logFC	*p* value*
Esophageal varices	hsa-miR-200a	0.67	4.85E-02
	hsa-miR-339-5p	0.65	4.75E-02
	hsa-miR-188-5p	−0.61	3.95E-02
Cholangiocarcinoma	hsa-let-7e	0.93	2.27E-02
	hsa-miR-124	1.78	2.16E-02
	hsa-miR-133a	1.71	2.17E-02
	hsa-miR-502-5p	−1.32	2.20E-02
Liver failure	hsa-let-7b	0.71	2.97E-02
	hsa-let-7d-star	0.83	2.50E-02
	hsa-miR-132	0.62	2.70E-02
	hsa-miR-133a	1.65	1.45E-02
	hsa-miR-141	1.43	8.94E-02
	hsa-mir-200a	1.37	1.61E-02
	hsa-mir-200b	1.42	1.00E-02
	hsa-mir-296-5p	1.06	1.63E-02
	hsa-mir-362-5p	1.21	1.14E-02
	hsa-mir-7	1.34	8.54E-02
	hsa-mir-765	0.78	4.94E-02
	hsa-mir-199a-5p	−0.73	4.85E-02
	hsa-mir-27b	−0.59	3.02E-02
	hsa-mir-374a	−0.88	1.26E-02
	hsa-mir-374b	−0.73	1.40E-02
	hsa-mir-376a	−1.29	2.09E-02
	hsa-mir-376c	−1.27	2.05E-02
	hsa-mir-584	−0.85	1.48E-02

* Level of significance: p <0.05 (moderated t test by LIMMA).

miRNAs (11 showed increased concentrations and seven showed decreased concentrations) (Table 4; Fig. 3A). Among the affected miRNAs, the levels of miR-133a (3.13-fold, p = 1.45E-02), miR-141 (2.70-fold, p = 8.94E-03), miR-200a (2.59-fold, p = 1.61E-03), miR-200b (2.67-fold, p = 1.00E-02), miR-296-5p (2.08-fold, p = 1.63E-02), miR-362-5p (2.31-fold, p = 1.14E-02), and miR-7 (2.53-fold, p = 8.54E-04) increased more than twofold, while the levels of miR-376a (0.41-folds, p=2.09E-02) and miR-376c (0.41-folds, p=2.05E-02) decreased the most.

The ability of affected miRNAs to predict liver failure was also evaluated. Three top-scoring pairs of six miRNAs were identified that can predict liver failure (AUC = 0.9879; Fig. 3B). However, due to the small sample size (n = 5), this observation needs to be verified with a larger sample.

To investigate the biological pathways associated with DE-miRNAs identified in serum samples of patients who later developed liver failure, pathway enrichment analysis was performed (Table S4). Based on the enriched pathways and the miRNA–target interactions (obtained from miRTarBase version 7.0)[23] (Table S5), we constructed a perturbed hypothetical network in patients who later developed liver failure reflected by their altered serum miRNA signature (Fig. 4). The network analysis revealed that miRNAs that showed increased concentration were involved in cell survival responses, such as inflammation (*e.g.* JAK-STAT and NF-κB), proliferation (*e.g.* GRB2 and RAF1), and apoptosis (*e.g.* BCL2, BAX, and CASP3/8/9) and

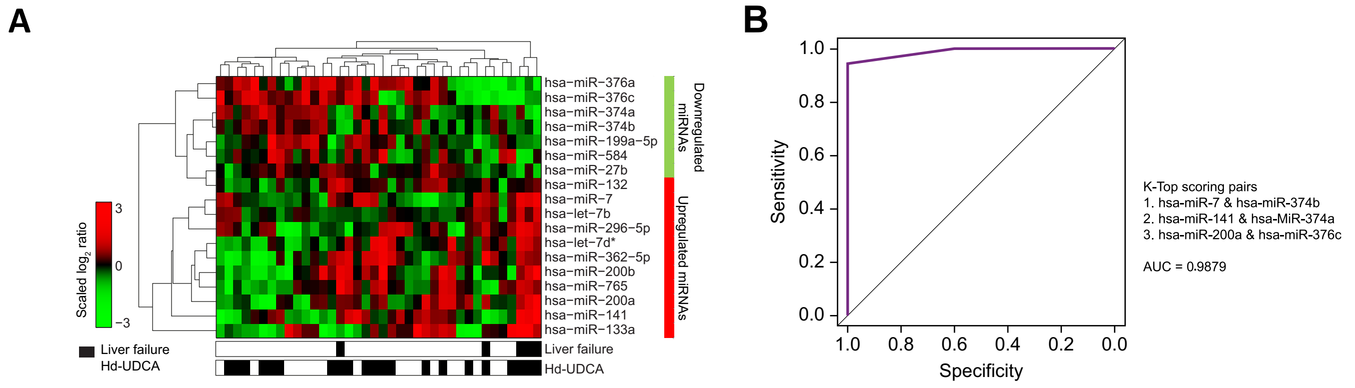

Fig. 3. Serum miRNA signatures associated with treatment outcome. (A) Unsupervised hierarchical cluster demonstrating the concentration differences of liver failure-associated miRNA signatures in 37 patients at post-entry time points. Red represents increased concentrations; green represents decreased concentrations. (B) The ROC curve indicates the sensitivity and specificity of three top-scoring miRNA pairs as a signature to identify patients with liver failure after hd-UDCA treatment. hd-UDCA, high-dose ursodeoxycholic acid; ROC, receiver operating characteristic.

miRNAs with decreased concentrations were involved in the cell cycle (*e.g.* CDK2/8, CDKN1A, and CKDN2B), fibrosis (*e.g.* TGFB2, TGFBR1, and SMAD2/3/4), and sterol biosynthesis (*e.g.* UGT2B15 and UGT2B17).

Discussion

It has been previously recognised that patients with PSC have distinct miRNAs in their serum and bile,[24] although these have not been evaluated longitudinally or in the context of ursodiol

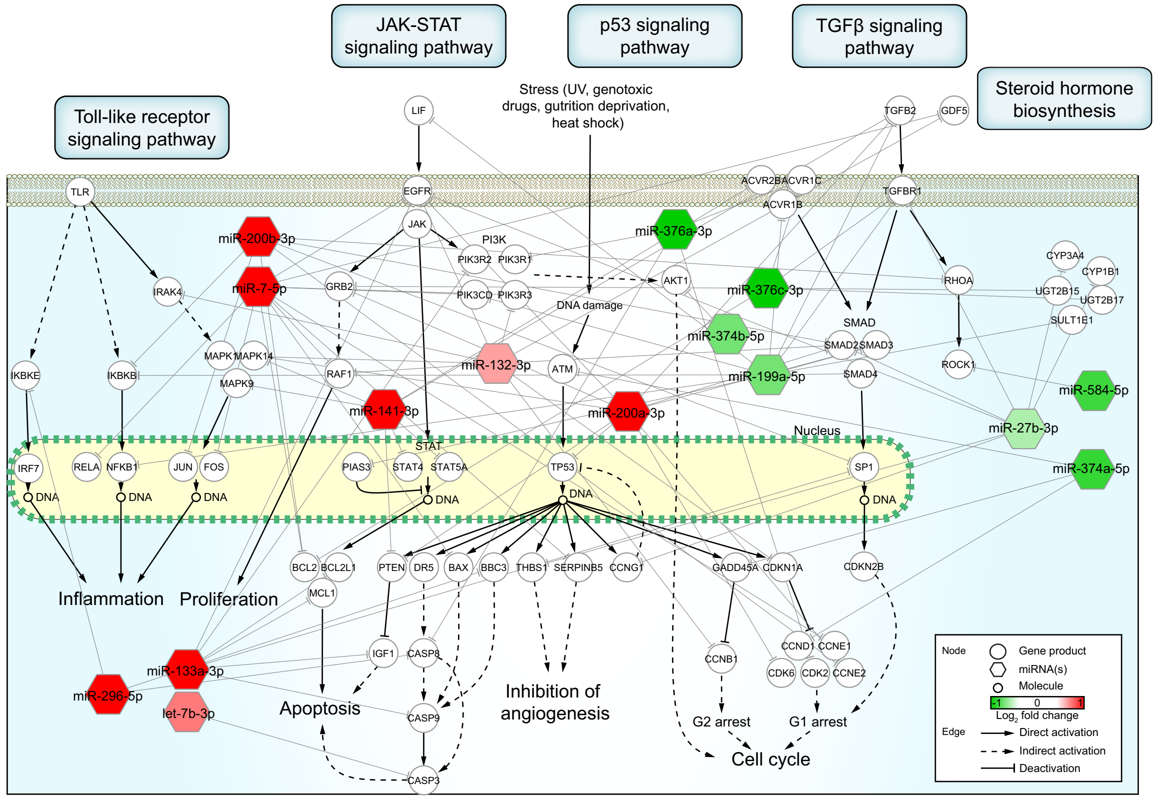

Fig. 4. Signaling pathways affected by miRNA changes associated with hd-UDCA treatment; potential mechanisms for liver-related adverse events. KEGG pathways were identified by pathway enrichment analysis using serum miRNAs altered in liver failure. Five key pathways were selected and highlighted in the network. The serum miRNAs were connected to their target genes in the pathways using miRTarBase, which is an experimentally validated interaction database. Circles and hexagons represent gene products and miRNAs, respectively. Red and green hexagons indicate up- and downregulation of miRNA in samples with liver failure, respectively. Solid and dashed arrows show direct and indirect interaction, respectively. Arrowheads or blocked lines denote activation or inhibition, respectively. DE, differentially expressed; hd-UDCA, high-dose ursodeoxycholic acid; KEGG, Kyoto Encyclopedia of Genes and Genomes; TGF, transforming growth factor.

therapy. Our study provides unique insight into miRNA serum profiles in patients with PSC and demonstrates changes over time observed with hd-UDCA treatment. To our knowledge, this is the only report of longitudinal changes in miRNA profiles in patients with PSC and in those treated with hd-UDCA compared with placebo.

Our data showed hd-UDCA treatment-related changes in miRNAs associated with immune-mediated and inflammation-related responses (*e.g.* antigen processing and presentation, complement and coagulation cascades, B/T cell activation, TGFβ signaling pathways, etc.). The alteration in miRNA and cytokine profiles in PSC identified in our cohort may provide a useful platform for further research of these markers in understanding disease pathogenesis in PSC.

In addition, we were able to analyse changes in miRNA patterns in patients receiving either placebo or hd-UDCA, both at specific time points and longitudinally. When analysing only the placebo-treated group at early *vs.* late time points, we found an increased number of differentially expressed miRNAs, suggesting that serum miRNA profiles evolve during the natural course of the disease. Moreover, a larger proportion of differentially expressed miRNAs was identified in patients treated with hd-UDCA, Among these miRNAs, only the concentration of miR-92b increased with hd-UDCA treatment, which, interestingly, has previously been shown to be an oncogene,[25] specifically via inhibition of cell cycle checkpoint gene *p57*.[26] Of the seven miRNAs the concentrations of which decreased, miR-885-5p, miR-122, and miR-194 have previously been observed to be upregulated in PSC and CC, as well as in other liver diseases.[27] This finding may again suggest a tumor suppressor role for these miRNAs via CDK2 and MCM5,[28] which is enhanced early in PSC progression and may become exhausted later in disease or when the condition is exacerbated by hd-UDCA treatment. miR-375, another miRNA with a decreased concentration in all patients treated with UDCA, has previously been found to be downregulated in fibrolamellar carcinoma.[29] miR-99a and miR-125b are known to be downregulated in CC[30] and are on the same cluster on chromosome 21q21.[31] Further mechanistic analysis would need to confirm which of these candidates has a key role in PSC pathogenesis, paying close attention to the temporal onset and magnitude of concentration changes to determine the overall diagnostic or prognostic value of these serum miRNAs. Overall, our findings highlight that, along with the previous description of increased disease severity and development of portal hypertension, unique differential changes in miRNA were observed in patients treated with hd-UDCA.

A better understanding of the relationship between changes in serum miRNA profiles and clinical course of PSC may contribute to a better understanding of disease mechanisms and aid the development of therapies for this liver disease. Increased neoplasia was previously observed among patients treated with hd-UDCA, suggesting that the latter induces dysregulation of cell proliferation or cell death.[32] Interestingly, this observation was consistent with the hd-UDCA treatment-associated serum miRNA patterns identified in this study (Fig. 2A,B). In the hd-UDCA group, we observed decreased levels of antiproliferation miRNAs, such as miR-125b.[33] Furthermore, miR-122 concentration in serum was significantly decreased in the hd-UDCA group compared with the placebo-group (Fig. 2A,B); an increased miR-122 level in blood may indicate increased liver injury.[34] There are unique opportunities through the study and utilisation of miRNA for prognostication and therapeutic intervention in hepatobiliary disease,[35] and our work further enhances the need for more in-depth study of miRNAs in PSC.

Additional strengths of our study are that it was derived from the only randomised control trial evaluating hd-UDCA *vs.* placebo in patients with PSC, and the only longitudinal data set evaluating miRNA changes in these patients. Limitations of this study include the relatively small sample size and lack of individual patient data; we hope that these findings may serve as a stepping-stone for a larger clinical trial that may further validate our findings, bringing cell-free blood miRNA biomarkers closer to clinical applications.

We also acknowledge that the spectrum of cell-free circulating miRNA, similar to all other biomolecules in the blood, such as proteins, is affected by many factors, including treatment as well as disease state. This is the foundation of using the changes of certain blood biomolecules, including specific miRNA species, as biomarkers for disease diagnosis, therapeutic efficacy, and others. Biological and technical factors can also affect the spectrum of cell-free miRNA. These include gender, age, blood sampling time, ample preparation methods, storage conditions, measurement methods, and others, which is why we previously highlighted the importance of standardisation for analysing cell-free RNA.[36] Nevertheless, the miRNA changes identified in this study were determined using the same measurement and sample preparation methods. Even though other factors might affect the spectrum of circulating miRNA, the major contribution factors are the disease state and treatment difference.

In conclusion, we identified unique longitudinal changes in serum miRNA profiles in patients with PSC and alterations of several miRNA concentrations in patients treated with hd-UDCA. Our study shows that hd-UDCA treatment contributes to a unique alteration in miRNA in patients with PSC that is distinctly different at both cross-sectional and longitudinal time points. Further investigation is warranted to determine whether these changes in miRNA concentrations are directly caused by treatment with hd-UDCA, which in turn then causes increased liver-related adverse events, or whether the hd-UDCA may be contributing to direct liver injury secondary to bile acid toxicity. Future studies examining miRNA changes in patients treated with standard-dose UDCA are also needed, because recent guidelines have endorsed its use.[37]

Abbreviations

ALP, alkaline phosphatase; AST, aspartate transaminase; cDNA, complementary DNA; ct, cycle threshold; EV, esophageal varices; FGF, fibroblast growth factor; HCC, hepatocellular cancer; hd-UDCA, high-dose ursodeoxycholic acid; NAFLD, non-alcoholic fatty liver disease; NGS, next-generation sequencing; ROC, receiver operating characteristic; PSC, primary sclerosing cholangitis; qRT-PCR, reverse transcription-PCR; SAE, serious adverse event; TGF, transforming growth factor.

Financial support

This study was supported by National Institutes of Health grants 3R01DK056924-08S1, 5K24DK002957, and 1R21HL112678.

Conflicts of interest

The authors declare no conflicts of interest that pertain to this work.

Please refer to the accompanying ICMJE disclosure forms for further details.

Authors' contributions

JTH, AS, and EG reviewed the literature and drafted the article. PH, BDM, T-KK, KW, YL, JBR, and JEN were involved in performing the experiments and statistical analysis for the study. KDL, NFL, and KVK designed the study, revised the manuscript for intellectual content, and were involved in the final approval of the publication. All authors agree to be accountable for their aspects of the work.

Data availability statement

The data that support the findings of this study are available from the corresponding author, KVK, upon reasonable request. The design of the study has been previously published and the information is provided in the Materials and Methods of that publication.

Acknowledgements

We thank Felicity T. Enders, Jody Moony, and Jill C. Keach for assistance.

Supplementary data

Supplementary data to this article can be found online at https://doi.org/10.1016/j.jhepr.2023.100729.

References

Author names in bold designate shared co-first authorship.

[1] Lindor KD, Kowdley KV, Harrison ME, American College of Gastroenterology. ACG clinical guideline: primary sclerosing cholangitis. Am J Gastroenterol 2015;110:646–659.

[2] Lindor KD, Kowdley KV, Luketic VA, Harrison ME, McCashland T, Befeler AS, et al. High-dose ursodeoxycholic acid for the treatment of primary sclerosing cholangitis. Hepatology 2009;50:808–814.

[3] Poy MN, Hausser J, Trajkovski M, Braun M, Collins S, Rorsman P, et al. miR-375 maintains normal pancreatic α- and β-cell mass. Proc Natl Acad Sci USA 2009;106:5813–5818.

[4] **Rayner KJ**, **Suárez Y**, Dávalos A, Parathath S, Fitzgerald ML, Tamehiro N, et al. MiR-33 contributes to the regulation of cholesterol homeostasis. Science 2010;328:1570–1573.

[5] Castoldi M, Vujic Spasic M, Altamura S, Elmén J, Lindow M, Kiss J, et al. The liver-specific microRNA miR-122 controls systemic iron homeostasis in mice. J Clin Invest 2011;121:1386–1396.

[6] Padgett KA, Lan RY, Leung PC, Lleo A, Dawson K, Pfeiff J, et al. Primary biliary cirrhosis is associated with altered hepatic microRNA expression. J Autoimmun 2009;32:246–253.

[7] Cheung O, Puri P, Eicken C, Contos MJ, Mirshahi F, Maher JW, et al. Nonalcoholic steatohepatitis is associated with altered hepatic MicroRNA expression. Hepatology 2008;48:1810–1820.

[8] Jopling C, Yi M, Lancaster A, Lemon S, Sarnow P. Modulation of hepatitis C virus RNA abundance by a liver-specific microRNA. Science 2005;309:1577–1581.

[9] Zhang X, Zhang E, Ma Z, Pei R, Jiang M, Schlaak JF, et al. Modulation of hepatitis B virus replication and hepatocyte differentiation by MicroRNA-1. Hepatology 2011;53:1476–1485.

[10] Tsai W-C, Hsu S-D, Hsu C-S, Lai T-C, Chen S-J, Shen R, et al. MicroRNA-122 plays a critical role in liver homeostasis and hepatocarcinogenesis. J Clin Invest 2012;122:2884–2897.

[11] Arroyo JD, Chevillet JR, Kroh EM, Ruf IK, Pritchard CC, Gibson DF, et al. Argonaute2 complexes carry a population of circulating microRNAs independent of vesicles in human plasma. Proc Natl Acad Sci USA 2011;108:5003–5008.

[12] **Mitchell PS**, **Parkin RK**, Kroh EM, Fritz BR, Wyman SK, Pogosova-Agadjanyan EL, et al. Circulating microRNAs as stable blood-based markers for cancer detection. Proc Natl Acad Sci USA 2008;105:10513–10518.

[13] Kroh EM, Parkin RK, Mitchell PS, Tewari M. Analysis of circulating microRNA biomarkers in plasma and serum using quantitative reverse transcription-PCR (qRT-PCR). Methods 2010;50:298–301.

[14] **Povero D**, **Tameda M**, Eguchi A, Ren W, Kim J, Myers R, et al. Protein and miRNA profile of circulating extracellular vesicles in patients with primary sclerosing cholangitis. Sci Rep 2022;12:3027.

[15] Li Y, Kowdley KV. Method for microRNA isolation from clinical serum samples. Anal Biochem 2012;431:69–75.

[16] Hastie T, Tibshirani R, Narasimhan B, Chu G. Impute: Imputation for microarray data. R package version 1.56.0. https://git.bioconductor.org/packages/impute Accessed 26 March 2023.

[17] Ritchie ME, Phipson B, Wu D, Hu Y, Law CW, Shi W, et al. LIMMA powers differential expression analyses for RNA-sequencing and microarray studies. Nucleic Acids Res 2015;43:e47.

[18] Backes C, Khaleeq QT, Meese E, Keller A. miEAA: microRNA enrichment analysis and annotation. Nucleic Acids Res 2016;44:W110–W116.

[19] Afsari B, Fertig EJ, Geman D, Marchionni L. switchBox: an R package for k-Top Scoring Pairs classifier development. Bioinformatics 2015;31(2):273–274.

[20] **Hu J**, **Chen C**, Liu Q, Liu B, Song C, Zhu S, et al. The role of the miR-31/FIH1 pathway in TGF-β-induced liver fibrosis. Clin Sci (Lond) 2015;129:305–317.

[21] **Zhuang LK**, **Yang YT**, **Ma X**, **Han B**, **Wang ZS**, **Zhao QY**, et al. MicroRNA-92b promotes hepatocellular carcinoma progression by targeting Smad7 and is mediated by long non-coding RNA XIST. Cell Death Dis 2016;7:e2203.

[22] Gui J, Tian Y, Wen X, Zhang W, Zhang P, Gao J, et al. Serum microRNA characterization identifies miR-885-5p as a potential marker for detecting liver pathologies. Clin Sci (Lond) 2011;120(5):183–193.

[23] Chou CH, Shrestha S, Yang CD, Chang NW, Lin YL, Liao KW, et al. miRTarBase update 2018: a resource for experimentally validated microRNA-target interactions. Nucleic Acids Res 2018;46:D296–D302.

[24] Voigtländer T, Gupta SK, Thum S, Fendrich J, Manns MP, Lankisch TO, et al. MicroRNAs in serum and bile of patients with primary sclerosing cholangitis and/or cholangiocarcinoma. PLoS One 2015;10:e0139305.

[25] **Wu ZB**, **Cai L**, Lin SJ, Lu JL, Yao Y, Zhou LF. The miR-92b functions as a potential oncogene by targeting on Smad3 in glioblastomas. Brain Res 2013;1529:16–25.

[26] Sengupta S, Nie J, Wagner RJ, Yang C, Stewart R, Thomson JA. MicroRNA 92b controls the G1/S checkpoint gene p57 in human embryonic stem cells. Stem Cells 2009;27:1524–1528.

[27] Bernuzzi F, Marabita F, Lleo A, Carbone M, Mirolo M, Marzioni M, et al. Serum microRNAs as novel biomarkers for primary sclerosing cholangitis and cholangiocarcinoma. Clin Exp Immunol 2016;185:61–71.

[28] Afanasyeva EA, Mestdagh P, Kumps C, Vandesompele J, Ehemann V, Theissen J, et al. MicroRNA miR-885-5p targets CDK2 and MCM5, activates p53 and inhibits proliferation and survival. Cell Death Differ 2011;18:974–984.

[29] **Dinh TA**, **Jewell ML**, Kanke M, Francisco A, Sritharan R, Turnham RE, et al. MicroRNA-375 suppresses the growth and invasion of fibrolamellar carcinoma. Cell Mol Gastroenterol Hepatol 2019;7:803–817.

[30] **Lin KY**, **Ye H**, Han BW, Wang WT, Wei PP, He B, et al. Genome-wide screen identified let-7c/miR-99a/miR-125b regulating tumor progression and stem-like properties in cholangiocarcinoma. Oncogene 2016;35:3376–3386.

[31] Sun D, Layer R, Mueller AC, Cichewicz MA, Negishi M, Paschal BM, et al. Regulation of several androgen-induced genes through the repression of the miR-99a/let-7c/miR-125b–2 miRNA cluster in prostate cancer cells. Oncogene 2014;33:1448–1457.

[32] Eaton JE, Silveira MG, Pardi DS, Sinakos E, Kowdley KV, Luketic VA, et al. High-dose ursodeoxycholic acid is associated with the development of colorectal neoplasia in patients with ulcerative colitis and primary sclerosing cholangitis. Am J Gastroenterol 2011;106:1638–1645.

[33] Liang L, Wong C-M, Ying Q, Fan DN-Y, Huang S, Ding J, et al. MicroRNA-125b suppressed human liver cancer cell proliferation and metastasis by directly targeting oncogene LIN28B2. Hepatology 2010;52:1731–1740.

[34] Wang K, Zhang S, Marzolf B, Troisch P, Brightman A, Hu Z, et al. Circulating microRNAs, potential biomarkers for drug-induced liver injury. Proc Natl Acad Sci USA 2009;106:4402–4407.

[35] Li Y, Kowdley KV. MicroRNAs in common human diseases. Genomics Proteomics Bioinformatics 2012;10:246–253.

[36] Lee I, Baxter D, Lee MY, Scherler K, Wang K. The importance of standardization on analyzing circulating RNA. Mol Diagn Ther 2017;21:259–268.

[37] Bowlus CL, Arrivé L, Bergquist A, Deneau M, Forman L, Ilyas S, et al. AASLD practice guidance on primary sclerosing cholangitis and cholangiocarcinoma. Hepatology 2023;77(2):659–702. https://doi.org/10.1002/hep.32771.

Journal of Hepatology, Volume *5*

Supplemental information

Serum miRNA profiles are altered in patients with primary sclerosing cholangitis receiving high-dose ursodeoxycholic acid

Jessica T. Hochberg, Aalam Sohal, Priya Handa, Bryan D. Maliken, Take-Kyun Kim, Kai Wang, Eric Gochanour, Yu Li, J. Bart Rose, James E. Nelson, Keith D. Lindor, Nicholas F. LaRusso, and Kris V. Kowdley

Serum miRNA profiles are altered in patients with primary sclerosing cholangitis receiving high-dose ursodeoxycholic acid

Jessica T. Hochberg, Aalam Sohal, Priya Handa, Bryan D.Maliken, Take-Kyun Kim, Kai Wang, Eric Gochanour, Yu Li, J Bart Rose, James E. Nelson[4], Keith D Lindor, Nicholas F. LaRusso, Kris V.Kowdley

Table of contents

1. Table S1
2. Table S2
3. Table S3
4. Table S4
5. Table S5

Table S1. Differentially expressed miRNAs at later time points in placebo or hd-UDCA

miRNA	LateA vs Placebo		LateB vs Placebo		LateA vs hd-UDCA		LateB vs hd-UDCA	
	logFC	p-Value	logFC	p-Value	logFC	p-Value	logFC	p-Value
hsa-let-7a	0.24	0.28	0.87	0.00	0.45	0.07	0.50	0.03
hsa-let-7d	0.34	0.16	0.83	0.00	0.43	0.06	0.70	0.00
hsa-let-7d*	-0.68	0.01	-0.40	0.17	-0.20	0.51	-0.13	0.67
hsa-let-7e	0.61	0.11	0.85	0.03	0.45	0.17	-0.13	0.70
hsa-let-7f	0.27	0.33	0.93	0.00	0.24	0.37	0.78	0.01
hsa-let-7i	0.10	0.67	0.39	0.09	0.17	0.40	0.88	0.00
hsa-miR-100	0.21	0.55	-0.24	0.55	-0.80	0.04	-1.15	0.01
hsa-miR-122	0.50	0.19	-0.19	0.67	-1.00	0.02	-1.67	0.00
hsa-miR-125b	0.27	0.34	-0.57	0.06	-0.68	0.01	-1.26	0.00
hsa-miR-142-3p	0.14	0.61	0.70	0.02	0.51	0.06	0.35	0.19
hsa-miR-146b-5p	-0.08	0.74	-0.19	0.46	-0.03	0.91	-0.62	0.01
hsa-miR-148a	-0.06	0.79	-0.37	0.12	-0.57	0.04	-1.06	0.00
hsa-miR-150	-0.02	0.95	-0.40	0.20	-0.42	0.09	-0.70	0.01
hsa-miR-152	-0.19	0.38	-0.23	0.30	-0.37	0.13	-0.71	0.01
hsa-miR-16	-0.08	0.71	-0.05	0.83	-0.03	0.91	0.74	0.01
hsa-miR-17	0.17	0.57	0.05	0.88	0.13	0.60	0.62	0.02
hsa-miR-182	-0.12	0.73	0.49	0.18	0.46	0.12	0.65	0.03
hsa-miR-185	0.04	0.86	-0.09	0.74	0.15	0.63	0.88	0.01
hsa-miR-18b	0.09	0.70	-0.24	0.33	0.39	0.12	0.62	0.02
hsa-miR-191	0.25	0.21	0.60	0.01	0.21	0.32	0.34	0.12
hsa-miR-192	0.36	0.32	-0.93	0.01	-0.53	0.15	-0.83	0.04
hsa-miR-193a-5p	0.11	0.68	-0.12	0.70	-0.45	0.23	-1.07	0.00
hsa-miR-193b	0.15	0.71	-0.51	0.22	-0.42	0.38	-1.09	0.04
hsa-miR-194	0.14	0.62	-0.54	0.09	-0.99	0.00	-0.88	0.02
hsa-miR-199b-5p	-0.26	0.52	1.11	0.01	-0.13	0.70	-0.32	0.40
hsa-miR-19a	-0.02	0.93	-0.08	0.72	0.22	0.25	0.75	0.00
hsa-miR-20a	0.08	0.69	-0.10	0.65	0.03	0.90	0.61	0.01
hsa-miR-20b	-0.40	0.22	-0.51	0.14	0.15	0.72	1.02	0.02
hsa-miR-214	-0.34	0.37	-0.81	0.05	-0.09	0.82	-0.65	0.10
hsa-miR-215	0.34	0.34	-0.68	0.07	-0.78	0.04	-0.92	0.04
hsa-miR-221	0.24	0.33	0.67	0.01	0.44	0.12	0.22	0.47
hsa-miR-23b	-0.11	0.52	0.03	0.84	-0.11	0.58	-0.64	0.00
hsa-miR-25	-0.12	0.55	-0.16	0.45	0.12	0.64	0.78	0.00
hsa-miR-26a	0.06	0.81	0.74	0.00	0.14	0.51	0.11	0.60
hsa-miR-26b	0.15	0.39	0.60	0.00	-0.08	0.69	0.30	0.17
hsa-miR-27b	0.02	0.92	-0.14	0.53	-0.04	0.84	-0.85	0.00
hsa-miR-296-5p	-0.18	0.60	-0.71	0.04	-0.09	0.74	0.32	0.21
hsa-miR-29a	0.18	0.58	-0.36	0.25	-0.39	0.11	-0.93	0.00
hsa-miR-30a	0.14	0.68	-0.34	0.37	-0.50	0.14	-0.85	0.02
hsa-miR-31	-0.31	0.43	-1.60	0.00	0.95	0.03	-0.54	0.23
hsa-miR-329	-0.14	0.70	-0.34	0.38	0.76	0.03	-0.23	0.52
hsa-miR-339-5p	0.03	0.93	0.70	0.11	0.86	0.01	0.36	0.30
hsa-miR-33a	0.11	0.71	0.68	0.04	0.41	0.16	0.40	0.18
hsa-miR-345	-0.05	0.83	-0.82	0.00	0.19	0.46	-0.89	0.00
hsa-miR-34a	1.07	0.02	0.02	0.97	-0.33	0.48	-0.17	0.72
hsa-miR-373-star	-0.51	0.30	-1.89	0.00	0.63	0.14	-1.05	0.01
hsa-miR-374b	0.46	0.12	1.03	0.00	0.35	0.21	0.29	0.30
hsa-miR-375	0.29	0.59	-0.17	0.76	-1.08	0.01	-1.31	0.02

hsa-miR-378	0.00	0.99	-0.31	0.17	-0.50	0.05	-0.76	0.01
hsa-miR-423-3p	0.28	0.17	0.49	0.03	0.59	0.00	0.10	0.61
hsa-miR-424	-0.69	0.01	-0.22	0.37	-0.19	0.38	-0.48	0.02
hsa-miR-433	-0.07	0.85	-0.15	0.71	0.89	0.01	0.15	0.66
hsa-miR-486-5p	-0.09	0.77	-0.06	0.83	0.23	0.46	1.46	0.00
hsa-miR-513a-5p	-0.01	0.98	-0.57	0.15	0.40	0.30	-0.79	0.04
hsa-miR-574-3p	0.43	0.09	-0.42	0.11	-0.19	0.50	-0.68	0.02
hsa-miR-584	0.04	0.91	0.56	0.11	0.85	0.01	0.42	0.19
hsa-miR-589	0.23	0.51	-1.02	0.01	0.94	0.01	-0.62	0.11
hsa-miR-598	-0.29	0.40	-0.07	0.84	-0.48	0.14	-0.65	0.02
hsa-miR-622	-0.58	0.06	-1.13	0.01	0.09	0.82	-1.15	0.02
hsa-miR-628-3p	-0.85	0.01	0.20	0.53	0.42	0.20	0.04	0.90
hsa-miR-629	-0.58	0.03	-0.13	0.64	0.70	0.02	0.42	0.15
hsa-miR-663	-0.25	0.45	-1.16	0.00	0.40	0.26	-0.46	0.17
hsa-miR-7	-0.12	0.75	-0.13	0.71	0.40	0.22	1.10	0.00
hsa-miR-885-5p	0.45	0.33	-0.16	0.75	-1.01	0.05	-1.85	0.00
hsa-miR-92b	-0.58	0.04	0.31	0.28	0.68	0.03	1.03	0.00
hsa-miR-93	0.17	0.43	-0.09	0.68	0.10	0.66	0.61	0.01
hsa-miR-99a	0.01	0.96	-0.55	0.10	-0.93	0.01	-1.61	0.00

Table S2. Differentially expressed miRNAs in hd-UDCA compared to Placebo

miRNA	hd-UDCA vs Placebo at Entry		hd-UDCA vs Placebo at Late A		hd-UDCA vs Placebo at Late B		Cluster
	logFC	p-Value	logFC	p-Value	logFC	p-Value	Number
hsa-miR-31	0.18	0.54	1.44	0.00	1.23	0.06	C1
hsa-miR-122	0.28	0.34	-1.21	0.01	-1.20	0.08	C2
hsa-miR-125b	0.22	0.27	-0.73	0.01	-0.47	0.19	C2
hsa-miR-493	0.67	0.04	1.31	0.01	0.10	0.83	C5
hsa-miR-130b	0.32	0.04	1.28	0.00	0.05	0.83	C1
hsa-miR-185	-0.29	0.16	-0.19	0.50	0.68	0.03	C3
hsa-miR-1	-0.09	0.78	1.26	0.02	-0.26	0.73	C1
hsa-miR-192	0.02	0.95	-0.87	0.03	0.11	0.80	C2
hsa-miR-194	0.30	0.16	-0.83	0.02	-0.04	0.93	C2
hsa-miR-199b-5p	-0.02	0.92	0.10	0.81	-1.45	0.00	C4
hsa-miR-19a	-0.23	0.09	0.01	0.97	0.60	0.04	C3
hsa-miR-20b	-0.57	0.04	-0.02	0.96	0.96	0.00	C3
hsa-miR-215	0.04	0.88	-1.08	0.01	-0.20	0.73	C2
hsa-miR-25	-0.33	0.05	-0.09	0.71	0.60	0.01	C3
hsa-miR-27a	0.05	0.69	0.24	0.33	-0.63	0.03	C4
hsa-miR-27b	0.08	0.58	0.02	0.93	-0.63	0.02	C4
hsa-miR-296-5p	-0.05	0.81	0.04	0.92	0.98	0.01	C3
hsa-miR-29b	-0.26	0.15	-0.83	0.03	0.30	0.14	C2
hsa-miR-339-5p	0.42	0.11	1.25	0.00	0.08	0.83	C1
hsa-miR-628-3p	-0.07	0.74	1.20	0.00	-0.23	0.44	C1
hsa-miR-433	0.19	0.45	1.16	0.01	0.49	0.16	C1
hsa-miR-92b	-0.13	0.51	1.13	0.01	0.59	0.05	C1
hsa-miR-887	0.32	0.20	1.10	0.01	0.72	0.11	C1
hsa-miR-34a	0.19	0.56	-1.21	0.02	0.00	1.00	C2
hsa-miR-363	-0.11	0.56	0.04	0.87	0.69	0.02	C3
hsa-miR-374b	0.14	0.48	0.03	0.92	-0.60	0.03	C4
hsa-miR-375	0.48	0.18	-0.89	0.03	-0.65	0.36	C2
hsa-miR-326	0.28	0.18	1.05	0.02	0.37	0.26	C1
hsa-miR-486-5p	-0.42	0.04	-0.11	0.76	1.10	0.00	C3
hsa-miR-185-star	-0.17	0.60	1.03	0.04	0.86	0.06	C1
hsa-miR-502-5p	0.58	0.02	1.01	0.01	0.14	0.82	C1
hsa-miR-627	0.24	0.29	0.98	0.01	0.24	0.50	C1
hsa-miR-490-3p	0.13	0.62	0.97	0.02	0.20	0.61	C1
hsa-miR-625*	0.12	0.59	0.89	0.03	-0.24	0.62	C1
hsa-miR-629	-0.39	0.04	0.89	0.02	0.16	0.65	C1
hsa-miR-146a	0.40	0.02	0.83	0.00	-0.42	0.17	C1
hsa-miR-346	0.53	0.13	0.76	0.04	0.16	0.85	C1
hsa-miR-7	-0.27	0.27	0.25	0.55	0.96	0.01	C3
hsa-miR-744	0.07	0.67	0.73	0.01	-0.12	0.72	C1
hsa-miR-885-5p	0.52	0.15	-0.93	0.04	-1.17	0.11	C2
hsa-miR-340	0.42	0.04	0.72	0.03	0.01	0.97	C1
hsa-miR-584	-0.10	0.65	0.71	0.03	-0.24	0.52	C1
hsa-miR-99a	0.28	0.24	-0.67	0.03	-0.78	0.06	C2

Table S3- Table S3. Pathways enriched by differentially expressed miRNAs in hd-UDCA compared to placebo. Mann-whiteny test was used to compare groups.

		p-Value					miRNA				
Pathway id	Pathway	C1	C2	C3	C4	C5	C1	C2	C3	C4	C5
hsa04062	Chemokine signaling pathway	0.02	0	1	1	1	hsa-miR-1-3p; hsa-miR-130b-3p; hsa-miR-146a-5p; hsa-miR-31-5p; hsa-miR-326; hsa-miR-339-5p; hsa-miR-346; hsa-miR-433-3p; hsa-miR-584-5p; hsa-miR-744-5p; hsa-miR-92b-3p	hsa-miR-122-5p; hsa-miR-125b-5p; hsa-miR-192-5p; hsa-miR-194-5p; hsa-miR-215-5p; hsa-miR-29b-3p; hsa-miR-34a-5p; hsa-miR-99a-5p			
hsa03030	DNA replication	0.05	0	1	1	1	hsa-miR-1-3p; hsa-miR-130b-3p; hsa-miR-31-5p; hsa-miR-339-5p; hsa-miR-744-5p	hsa-miR-125b-5p; hsa-miR-192-5p; hsa-miR-215-5p; hsa-miR-34a-5p; hsa-miR-885-5p			
hsa02010	ABC transporters	0.03	1	0.04	1	1	hsa-miR-1-3p; hsa-miR-339-5p; hsa-miR-346; hsa-miR-433-3p; hsa-miR-92b-3p		hsa-miR-19a-3p; hsa-miR-296-5p; hsa-miR-7-5p		
hsa04110	Cell cycle	1	0	0.03	1	1		hsa-miR-122-5p; hsa-miR-125b-5p; hsa-miR-192-5p; hsa-miR-194-5p; hsa-miR-215-5p; hsa-miR-29b-3p; hsa-miR-34a-5p; hsa-miR-885-5p; hsa-miR-99a-5p	hsa-miR-185-5p; hsa-miR-19a-3p; hsa-miR-20b-5p; hsa-miR-25-3p; hsa-miR-296-5p; hsa-miR-363-3p; hsa-miR-7-5p		
WP707	DNA damage response	1	0	0.01	1	1		hsa-miR-122-5p; hsa-miR-125b-5p; hsa-miR-192-5p; hsa-miR-215-5p; hsa-miR-29b-3p; hsa-miR-34a-5p;	hsa-miR-185-5p; hsa-miR-19a-3p; hsa-miR-20b-5p; hsa-miR-25-3p; hsa-miR-296-5p;		

								hsa-miR-885-5p; hsa-miR-99a-5p	hsa-miR-363-3p; hsa-miR-7-5p		
hsa04144	Endocytosis	1	0	0.05	1	1		hsa-miR-122-5p; hsa-miR-125b-5p; hsa-miR-192-5p; hsa-miR-194-5p; hsa-miR-215-5p; hsa-miR-29b-3p; hsa-miR-34a-5p; hsa-miR-99a-5p	hsa-miR-185-5p; hsa-miR-19a-3p; hsa-miR-20b-5p; hsa-miR-25-3p; hsa-miR-296-5p; hsa-miR-7-5p		
hsa04012	ErbB signaling pathway	1	0.01	0.03	1	1		hsa-miR-122-5p; hsa-miR-125b-5p; hsa-miR-192-5p; hsa-miR-194-5p; hsa-miR-215-5p; hsa-miR-34a-5p; hsa-miR-99a-5p	hsa-miR-185-5p; hsa-miR-19a-3p; hsa-miR-20b-5p; hsa-miR-296-5p; hsa-miR-363-3p; hsa-miR-7-5p		
hsa04630	Jak STAT signaling pathway	1	0.01	0.02	1	1		hsa-miR-122-5p; hsa-miR-125b-5p; hsa-miR-192-5p; hsa-miR-194-5p; hsa-miR-215-5p; hsa-miR-29b-3p; hsa-miR-34a-5p	hsa-miR-185-5p; hsa-miR-19a-3p; hsa-miR-20b-5p; hsa-miR-25-3p; hsa-miR-296-5p; hsa-miR-7-5p		
P00059	p53 pathway	1	0	0.01	1	1		hsa-miR-122-5p; hsa-miR-125b-5p; hsa-miR-192-5p; hsa-miR-194-5p; hsa-miR-215-5p; hsa-miR-29b-3p; hsa-miR-34a-5p; hsa-miR-885-5p; hsa-miR-99a-5p	hsa-miR-185-5p; hsa-miR-19a-3p; hsa-miR-20b-5p; hsa-miR-25-3p; hsa-miR-296-5p; hsa-miR-363-3p; hsa-miR-7-5p		
WP75	Toll Like Receptor signaling	1	0	0.04	1	1		hsa-miR-122-5p; hsa-miR-125b-5p; hsa-miR-192-5p; hsa-miR-194-5p; hsa-miR-215-5p; hsa-miR-29b-3p; hsa-miR-34a-5p	hsa-miR-185-5p; hsa-miR-19a-3p; hsa-miR-296-5p; hsa-miR-486-5p; hsa-miR-7-5p		
WP138	Androgen receptor signaling pathway	1	0.01	0.02	1	1		hsa-miR-122-5p; hsa-miR-125b-5p; hsa-miR-192-5p; hsa-miR-	hsa-miR-185-5p; hsa-miR-19a-3p; hsa-miR-20b-5p;		

								194-5p; hsa-miR-215-5p; hsa-miR-29b-3p; hsa-miR-34a-5p; hsa-miR-99a-5p	hsa-miR-25-3p; hsa-miR-296-5p; hsa-miR-363-3p; hsa-miR-7-5p		
WP236	Adipogenesis	1	0.01	0.03	0.02	1		hsa-miR-122-5p; hsa-miR-125b-5p; hsa-miR-192-5p; hsa-miR-215-5p; hsa-miR-29b-3p; hsa-miR-34a-5p; hsa-miR-99a-5p	hsa-miR-185-5p; hsa-miR-19a-3p; hsa-miR-20b-5p; hsa-miR-25-3p; hsa-miR-363-3p; hsa-miR-7-5p	hsa-miR-199b-5p; hsa-miR-27a-3p; hsa-miR-27b-3p; hsa-miR-374b-5p	
WP710	DNA damage response only ATM dependent	1	0.05	0.02	0.04	1		hsa-miR-122-5p; hsa-miR-125b-5p; hsa-miR-192-5p; hsa-miR-194-5p; hsa-miR-215-5p; hsa-miR-29b-3p; hsa-miR-34a-5p	hsa-miR-185-5p; hsa-miR-19a-3p; hsa-miR-20b-5p; hsa-miR-25-3p; hsa-miR-296-5p; hsa-miR-363-3p; hsa-miR-7-5p	hsa-miR-199b-5p; hsa-miR-27a-3p; hsa-miR-27b-3p; hsa-miR-374b-5p	
hsa04020	Calcium signaling pathway	1	0.01	1	0.05	1		hsa-miR-122-5p; hsa-miR-125b-5p; hsa-miR-192-5p; hsa-miR-215-5p; hsa-miR-34a-5p; hsa-miR-99a-5p		hsa-miR-199b-5p; hsa-miR-27a-3p; hsa-miR-27b-3p	
WP2034	Leptin signaling pathway	1	0	1	0.02	1		hsa-miR-122-5p; hsa-miR-125b-5p; hsa-miR-192-5p; hsa-miR-194-5p; hsa-miR-215-5p; hsa-miR-29b-3p; hsa-miR-34a-5p; hsa-miR-99a-5p		hsa-miR-199b-5p; hsa-miR-27a-3p; hsa-miR-27b-3p; hsa-miR-374b-5p	
hsa00562	Inositol phosphate metabolism	1	0	1	0.03	1		hsa-miR-122-5p; hsa-miR-125b-5p; hsa-miR-192-5p; hsa-miR-215-5p; hsa-miR-29b-3p; hsa-miR-34a-5p		hsa-miR-27a-3p; hsa-miR-27b-3p; hsa-miR-374b-5p	
P00046	Oxidative stress response	1	0	1	0.03	1		hsa-miR-122-5p; hsa-miR-125b-5p; hsa-miR-192-5p; hsa-miR-		hsa-miR-27a-3p; hsa-miR-27b-3p;	

								215-5p; hsa-miR-29b-3p; hsa-miR-34a-5p		hsa-miR-374b-5p	
P00033	Insulin IGF pathway protein kinase B signaling cascade	1	0	1	0.05	1		hsa-miR-122-5p; hsa-miR-125b-5p; hsa-miR-192-5p; hsa-miR-194-5p; hsa-miR-215-5p; hsa-miR-29b-3p; hsa-miR-34a-5p; hsa-miR-99a-5p		hsa-miR-27a-3p; hsa-miR-27b-3p; hsa-miR-374b-5p	
hsa04520	Adherens junction	1	0	1	1	1		hsa-miR-122-5p; hsa-miR-125b-5p; hsa-miR-192-5p; hsa-miR-194-5p; hsa-miR-215-5p; hsa-miR-29b-3p; hsa-miR-34a-5p; hsa-miR-99a-5p			
P00005	Angiogenesis	1	0.02	1	1	1		hsa-miR-122-5p; hsa-miR-125b-5p; hsa-miR-192-5p; hsa-miR-215-5p; hsa-miR-29b-3p; hsa-miR-34a-5p; hsa-miR-99a-5p			
hsa04612	Antigen processing and presentation	1	0.02	1	1	1		hsa-miR-122-5p; hsa-miR-125b-5p; hsa-miR-192-5p; hsa-miR-215-5p; hsa-miR-34a-5p			
hsa04610	Complement and coagulation cascades	1	0	1	1	1		hsa-miR-122-5p; hsa-miR-192-5p; hsa-miR-215-5p; hsa-miR-29b-3p; hsa-miR-99a-5p			
P00010	B cell activation	1	0	1	1	1		hsa-miR-122-5p; hsa-miR-125b-5p; hsa-miR-192-5p; hsa-miR-194-5p; hsa-miR-215-5p; hsa-miR-29b-3p; hsa-miR-34a-5p			

P00053	T cell activation	1	0	1	1	1		hsa-miR-122-5p; hsa-miR-125b-5p; hsa-miR-192-5p; hsa-miR-194-5p; hsa-miR-215-5p; hsa-miR-29b-3p; hsa-miR-34a-5p			
hsa04350	TGF beta signaling pathway	1	0.01	1	1	1		hsa-miR-122-5p; hsa-miR-125b-5p; hsa-miR-192-5p; hsa-miR-194-5p; hsa-miR-215-5p; hsa-miR-29b-3p; hsa-miR-34a-5p			
WP231	TNF alpha Signaling Pathway	1	0.02	1	1	1		hsa-miR-122-5p; hsa-miR-125b-5p; hsa-miR-192-5p; hsa-miR-194-5p; hsa-miR-215-5p; hsa-miR-34a-5p			
hsa04210	Apoptosis	1	0.03	1	1	1		hsa-miR-122-5p; hsa-miR-125b-5p; hsa-miR-192-5p; hsa-miR-215-5p; hsa-miR-29b-3p; hsa-miR-34a-5p			
hsa04512	ECM receptor interaction	1	0	1	1	1		hsa-miR-192-5p; hsa-miR-194-5p; hsa-miR-215-5p; hsa-miR-29b-3p; hsa-miR-34a-5p; hsa-miR-99a-5p			
P00014	Cholesterol biosynthesis	1	0.01	1	1	1		hsa-miR-125b-5p; hsa-miR-192-5p; hsa-miR-215-5p			
hsa00534	Heparan sulfate biosynthesis	1	0	1	1	1		hsa-miR-125b-5p; hsa-miR-192-5p; hsa-miR-215-5p; hsa-miR-34a-5p; hsa-miR-99a-5p			
hsa04010	MAPK signaling pathway	1	0.01	1	1	1		hsa-miR-122-5p; hsa-miR-125b-5p; hsa-miR-192-5p; hsa-miR-			

								194-5p; hsa-miR-215-5p; hsa-miR-29b-3p; hsa-miR-34a-5p; hsa-miR-99a-5p			
WP129	Matrix Metalloproteinases	1	0.01	1	1	1		hsa-miR-125b-5p; hsa-miR-192-5p; hsa-miR-215-5p; hsa-miR-29b-3p			
hsa04950	Maturity onset diabetes of the young	1	0.01	1	1	1		hsa-miR-192-5p; hsa-miR-215-5p; hsa-miR-34a-5p			
hsa04150	mTOR signaling pathway	1	0	1	1	1		hsa-miR-122-5p; hsa-miR-125b-5p; hsa-miR-192-5p; hsa-miR-215-5p; hsa-miR-29b-3p; hsa-miR-34a-5p; hsa-miR-99a-5p			
hsa04621	NOD like receptor signaling pathway	1	0.03	1	1	1		hsa-miR-122-5p; hsa-miR-125b-5p; hsa-miR-192-5p; hsa-miR-215-5p; hsa-miR-34a-5p			
WP623	Oxidative phosphorylation	1	0.03	1	1	1		hsa-miR-125b-5p; hsa-miR-192-5p; hsa-miR-215-5p; hsa-miR-99a-5p			
hsa03320	PPAR signaling pathway	1	0.04	1	1	1		hsa-miR-122-5p; hsa-miR-192-5p; hsa-miR-215-5p; hsa-miR-34a-5p			
hsa00600	Sphingolipid metabolism	1	0	1	1	1		hsa-miR-122-5p; hsa-miR-125b-5p; hsa-miR-192-5p; hsa-miR-215-5p; hsa-miR-34a-5p; hsa-miR-99a-5p			
hsa04370	VEGF signaling pathway	1	0.02	1	1	1		hsa-miR-122-5p; hsa-miR-125b-5p; hsa-			

								miR-194-5p; hsa-miR-29b-3p; hsa-miR-34a-5p; hsa-miR-99a-5p			
hsa04310	Wnt signaling pathway	1	0.02	1	1	1		hsa-miR-122-5p; hsa-miR-125b-5p; hsa-miR-192-5p; hsa-miR-194-5p; hsa-miR-215-5p; hsa-miR-29b-3p; hsa-miR-34a-5p			

Table S4- Pathways enriched by differentially expressed miRNAs in serum of patients who later developed liver failure

KEGG pathway	Up-regulated		Down-regulated	
	p-Value	miRNAs	p-Value	miRNAs
hsa00591 Linoleic acid metabolism	0.03	hsa-let-7b-5p; hsa-miR-132-3p		
hsa02010 ABC transporters	0.03	hsa-let-7b-5p; hsa-miR-132-3p; hsa-miR-296-5p		
hsa04012 ErbB signaling pathway	0.01	hsa-let-7b-5p; hsa-miR-132-3p; hsa-miR-141-3p; hsa-miR-200a-3p; hsa-miR-200b-3p; hsa-miR-296-5p		
hsa04114 Oocyte meiosis	0.04	hsa-let-7b-5p; hsa-miR-132-3p; hsa-miR-141-3p; hsa-miR-200a-3p; hsa-miR-200b-3p		
hsa04115 p53 signaling pathway	0.02	hsa-let-7b-5p; hsa-miR-132-3p; hsa-miR-141-3p; hsa-miR-200a-3p; hsa-miR-200b-3p; hsa-miR-296-5p		
hsa04310 Wnt signaling pathway	0.01	hsa-let-7b-5p; hsa-miR-132-3p; hsa-miR-141-3p; hsa-miR-200a-3p; hsa-miR-200b-3p; hsa-miR-296-5p	0.01	hsa-miR-199a-5p; hsa-miR-27b-3p; hsa-miR-374a-5p; hsa-miR-374b-5p; hsa-miR-584-5p
hsa04350 TGF beta signaling pathway	0.04	hsa-let-7b-5p; hsa-miR-132-3p; hsa-miR-141-3p; hsa-miR-200a-3p; hsa-miR-200b-3p	0.00	hsa-miR-199a-5p; hsa-miR-27b-3p; hsa-miR-374a-5p; hsa-miR-374b-5p; hsa-miR-584-5p
hsa04370 VEGF signaling pathway	0.02	hsa-let-7b-5p; hsa-miR-132-3p; hsa-miR-141-3p; hsa-miR-200a-3p; hsa-miR-200b-3p		
hsa04620 Toll like receptor signaling pathway	0.02	hsa-let-7b-5p; hsa-miR-132-3p; hsa-miR-141-3p; hsa-miR-200a-3p; hsa-miR-296-5p		
hsa04621 NOD like receptor signaling pathway	0.05	hsa-let-7b-5p; hsa-miR-141-3p; hsa-miR-200a-3p; hsa-miR-200b-3p		
hsa04622 RIG I like receptor signaling pathway	0.02	hsa-let-7b-5p; hsa-miR-141-3p; hsa-miR-200a-3p; hsa-miR-296-5p		
hsa04650 Natural killer cell mediated cytotoxicity	0.01	hsa-let-7b-5p; hsa-miR-132-3p; hsa-miR-141-3p; hsa-miR-200a-3p; hsa-miR-200b-3p		
hsa04710 Circadian rhythm mammal	0.04	hsa-let-7b-5p; hsa-miR-141-3p		
hsa04722 Neurotrophin signaling pathway	0.00	hsa-let-7b-5p; hsa-miR-132-3p; hsa-miR-141-3p; hsa-miR-200a-3p; hsa-miR-200b-3p; hsa-miR-296-5p; hsa-miR-765		
hsa04916 Melanogenesis	0.01	hsa-let-7b-5p; hsa-miR-132-3p; hsa-miR-141-3p; hsa-miR-200a-3p; hsa-miR-200b-3p	0.02	hsa-miR-199a-5p; hsa-miR-27b-3p; hsa-miR-374a-5p; hsa-miR-374b-5p
hsa05014 Amyotrophic lateral sclerosis ALS	0.05	hsa-miR-141-3p; hsa-miR-200a-3p; hsa-miR-200b-3p; hsa-miR-296-5p		
hsa05212 Pancreatic cancer	0.02	hsa-let-7b-5p; hsa-miR-132-3p; hsa-miR-141-3p; hsa-miR-200a-3p; hsa-miR-200b-3p; hsa-miR-296-5p		
hsa05213 Endometrial cancer	0.04	hsa-let-7b-5p; hsa-miR-141-3p; hsa-miR-200a-3p; hsa-miR-200b-3p; hsa-miR-296-5p		

hsa05214 Glioma	0.03	hsa-let-7b-5p; hsa-miR-132-3p; hsa-miR-141-3p; hsa-miR-200a-3p; hsa-miR-200b-3p; hsa-miR-296-5p		
hsa05215 Prostate cancer	0.05	hsa-let-7b-5p; hsa-miR-132-3p; hsa-miR-141-3p; hsa-miR-200a-3p; hsa-miR-200b-3p; hsa-miR-296-5p		
hsa05216 Thyroid cancer	0.01	hsa-let-7b-5p; hsa-miR-141-3p; hsa-miR-200a-3p; hsa-miR-200b-3p; hsa-miR-296-5p		
hsa05217 Basal cell carcinoma	0.02	hsa-miR-132-3p; hsa-miR-141-3p; hsa-miR-200a-3p; hsa-miR-200b-3p		
hsa05220 Chronic myeloid leukemia	0.03	hsa-let-7b-5p; hsa-miR-132-3p; hsa-miR-141-3p; hsa-miR-200a-3p; hsa-miR-200b-3p; hsa-miR-296-5p		
hsa05221 Acute myeloid leukemia	0.02	hsa-let-7b-5p; hsa-miR-141-3p; hsa-miR-200a-3p; hsa-miR-200b-3p; hsa-miR-296-5p		
hsa05222 Small cell lung cancer	0.02	hsa-let-7b-5p; hsa-miR-132-3p; hsa-miR-141-3p; hsa-miR-200a-3p; hsa-miR-200b-3p; hsa-miR-296-5p		
hsa05412 Arrhythmogenic right ventricular cardiomyopathy ARVC	0.04	hsa-let-7b-5p; hsa-miR-141-3p; hsa-miR-200a-3p; hsa-miR-200b-3p		
hsa00140 Steroid hormone biosynthesis			0.03	hsa-miR-199a-5p; hsa-miR-27b-3p
hsa00512 O Glycan biosynthesis			0.04	hsa-miR-27b-3p; hsa-miR-374b-5p
hsa04630 Jak STAT signaling pathway			0.04	hsa-miR-199a-5p; hsa-miR-27b-3p; hsa-miR-374a-5p; hsa-miR-374b-5p

Table S5. Experimentally validated interactions between DE-miRNAs in serum of patients who later developed liver failure and the target genes

miRNA	Target	Number of evidences	Number of publications	evidence	hsa04115 p53 signaling pathway	hsa04630 Jak STAT signaling pathway	hsa04620 Toll like receptor signaling pathway	hsa04350 TGF beta signaling pathway	hsa00140 Steroid hormone biosynthesis
hsa-let-7b-3p	CASP3	4	1	Immunohistochemistry//Luciferase reporter assay//qRT-PCR//Western blot	O				
hsa-let-7b-5p	CCND1	10	3	Immunoblot//Immunofluorescence//Luciferase reporter assay//qRT-PCR//Reporter assay;Other//immunoblot//Northern blot//Western blot//Microarray//Annexin V-FITC	O				
hsa-let-7b-5p	CCND2	4	2	Luciferase reporter assay//Immunohistochemistry//QRTPCR//Western blot	O				
hsa-let-7b-5p	CDK6	3	1	Luciferase reporter assay//Microarray//Western blot	O				
hsa-let-7b-5p	TNFRSF10B	2	1	qRT-PCR//Western blot	O				
hsa-let-7b-5p	ACVR1	3	1	Luciferase reporter assay//Microarray//qRT-PCR				O	
hsa-let-7b-5p	AKT2	3	1	Luciferase reporter assay//qRT-PCR//Western blot		O			
hsa-let-7b-5p	HRAS	3	1	Immunoblot//qRT-PCR//Western blot		O			
hsa-let-7b-5p	IFNB1	3	1	ELISA//Luciferase reporter assay//qRT-PCR		O			
hsa-let-7b-5p	PDGFRA	3	1	Luciferase reporter assay//Microarray//qRT-PCR		O			
hsa-let-7b-5p	TGFBR1	3	1	GFP reporter assay//Western blot//ELISA				O	
hsa-let-7b-5p	TLR4	1	1	Luciferase reporter assay			O		
hsa-miR-132-3p	CCNB1	1	1	Western blot;qRT-PCR	O				
hsa-miR-132-3p	CDKN1A	3	1	qRT-PCR//Luciferase reporter assay//Western blot	O				
hsa-miR-132-3p	EGFR	4	1	Immunohistochemistry//Luciferase reporter assay//qRT-PCR//Western blot		O			
hsa-miR-132-3p	GDF5	2	1	Luciferase reporter assay//Western blot				O	
hsa-miR-132-3p	IRAK4	1	1	Luciferase reporter assay			O		
hsa-miR-132-3p	MAPK1	4	1	Luciferase reporter assay//QRTPCR//Microarray//Western blot			O		

hsa-miR-132-3p	PIK3R3	3	1	Luciferase reporter assay//qRT-PCR//Western blot		O			
hsa-miR-132-3p	RAF1	4	1	Immunohistochemistry//Luciferase reporter assay//qRT-PCR//Western blot		O			
hsa-miR-132-3p	SMAD2	3	1	GFP reporter assay//qRT-PCR//Western blot				O	
hsa-miR-133a-3p	BCL2L1	4	2	Luciferase reporter assay//Western blot//ChIP-seq//qRT-PCR	O				
hsa-miR-133a-3p	CASP9	1	1	Luciferase reporter assay	O				
hsa-miR-133a-3p	IGF1	1	1	Luciferase reporter assay	O				
hsa-miR-133a-3p	EGFR	4	5	Luciferase reporter assay//Western blot//qRT-PCR//Flow		O			
hsa-miR-133a-3p	MCL1	4	2	Luciferase reporter assay//Western blot//ChIP-seq//qRT-PCR		O			
hsa-miR-133a-3p	PIK3R2	1	1	Reporter assay		O			
hsa-miR-133a-3p	SP1	3	2	Luciferase reporter assay//Immunofluorescence//Western blot				O	
hsa-miR-141-3p	PTEN	3	1	Luciferase reporter assay//Western blot//Reporter assay;Western blot;Other	O				
hsa-miR-141-3p	ACVR2B	2	1	Luciferase reporter assay//Western blot				O	
hsa-miR-141-3p	MAPK14	3	1	Luciferase reporter assay//Microarray//Western blot			O		
hsa-miR-141-3p	MAPK9	1	1	Western blot			O		
hsa-miR-141-3p	STAT4	4	1	Luciferase reporter assay//qRT-PCR//Western blot//Immunohistochemistry		O			
hsa-miR-141-3p	STAT5A	3	1	Immunocytochemistry//Western blot//Luciferase reporter assay		O			
hsa-miR-141-3p	TGFB2	5	2	Immunoblot//Luciferase reporter assay//qRT-PCR//Flow//Western blot				O	
hsa-miR-199a-5p	ACVR1B	4	1	Immunoprecipitaion//qRT-PCR//Western blot//Luciferase reporter assay				O	
hsa-miR-199a-5p	IKBKB	5	1	Reporter assay//Western blot//GFP reporter assay//Luciferase reporter assay//qRT-PCR			O		
hsa-miR-199a-5p	LIF	2	1	ELISA//Luciferase reporter assay		O			
hsa-miR-199a-5p	NFKB1	3	1	Luciferase reporter assay//qRT-PCR//Western blot			O		
hsa-miR-199a-5p	PIAS3	3	1	Luciferase reporter assay//qRT-PCR//Western blot		O			
hsa-miR-199a-5p	PIK3CD	2	1	Luciferase reporter assay//qRT-PCR		O			

hsa-miR-199a-5p	SMAD3	1	1	Luciferase reporter assay				O	
hsa-miR-199a-5p	SMAD4	1	2	Luciferase reporter assay				O	
hsa-miR-199a-5p	SULT1E1	1	1	Luciferase reporter assay					O
hsa-miR-199a-5p	TGFB2	2	1	Luciferase reporter assay//qRT-PCR				O	
hsa-miR-199a-5p	TGFBR1	4	1	Luciferase reporter assay//Microarray//qRT-PCR//Western blot				O	
hsa-miR-200a-3p	CCNE2	1	1	Luciferase reporter assay	O				
hsa-miR-200a-3p	CDK6	3	1	Luciferase reporter assay//qRT-PCR//Western blot	O				
hsa-miR-200a-3p	PTEN	2	1	qRT-PCR//Western blot	O				
hsa-miR-200a-3p	TP53	2	1	Luciferase reporter assay//Western blot	O				
hsa-miR-200a-3p	EGFR	1	1	Western blot		O			
hsa-miR-200a-3p	GRB2	1	1	Luciferase reporter assay		O			
hsa-miR-200a-3p	MAPK14	3	1	Luciferase reporter assay//Microarray//Western blot			O		
hsa-miR-200a-3p	SMAD2	1	1	Luciferase reporter assay				O	
hsa-miR-200a-3p	SMAD3	1	1	Luciferase reporter assay				O	
hsa-miR-200a-3p	TGFB2	3	1	Luciferase reporter assay//qRT-PCR//Western blot				O	
hsa-miR-200b-3p	BCL2	3	1	Luciferase reporter assay//qRT-PCR//Western blot	O				
hsa-miR-200b-3p	CCNE2	1	1	Luciferase reporter assay	O				
hsa-miR-200b-3p	IKBKB	3	1	Luciferase reporter assay//qRT-PCR//Western blot			O		
hsa-miR-200b-3p	JUN	3	1	Luciferase reporter assay//qRT-PCR//Western blot			O		
hsa-miR-200b-3p	RHOA	1	1	Luciferase reporter assay				O	
hsa-miR-200b-3p	SMAD2	1	1	Luciferase reporter assay				O	
hsa-miR-200b-3p	SP1	4	2	In situ hybridization//Luciferase reporter assay//qRT-PCR//Western blot				O	

hsa-miR-27b-3p	CCNG1	5	1	Immunohistochemistry//In situ hybridization//Luciferase reporter assay//qRT-PCR//Western blot	O				
hsa-miR-27b-3p	THBS1	1	1	Luciferase reporter assay	O				
hsa-miR-27b-3p	CYP1B1	5	2	Immunohistochemistry//Luciferase reporter assay//qRT-PCR//Western blot//Reporter assay;Other					O
hsa-miR-27b-3p	CYP3A4	2	1	Immunoblot//Luciferase reporter assay					O
hsa-miR-27b-3p	EGFR	3	1	qRT-PCR//Luciferase reporter assay//Western blot		O			
hsa-miR-27b-3p	SMAD2	2	1	Luciferase reporter assay//Western blot				O	
hsa-miR-27b-3p	TGFBR1	2	1	Luciferase reporter assay//Western blot				O	
hsa-miR-296-5p	BBC3	3	1	Luciferase reporter assay//qRT-PCR//Western blot	O				
hsa-miR-296-5p	CASP8	3	1	Immunoprecipitaion//Luciferase reporter assay//qRT-PCR	O				
hsa-miR-296-5p	IKBKE	1	1	Luciferase reporter assay			O		
hsa-miR-374a-5p	ATM	3	1	Immunoblot//Luciferase reporter assay//qRT-PCR	O				
hsa-miR-374a-5p	CCND1	2	1	Luciferase reporter assay//Western blot	O				
hsa-miR-374a-5p	GADD45A	3	1	Immunoblot//Luciferase reporter assay//qRT-PCR	O				
hsa-miR-374b-5p	AKT1	4	1	Luciferase reporter assay//Microarray//qRT-PCR//Western blot		O			
hsa-miR-376a-3p	CASP8	3	1	Luciferase reporter assay//qRT-PCR//Western blot	O				
hsa-miR-376a-3p	CDK2	1	1	Luciferase reporter assay	O				
hsa-miR-376a-3p	ACVR1C	2	1	Luciferase reporter assay//Reporter assay;Western blot				O	
hsa-miR-376a-3p	PIK3R1	3	1	Luciferase reporter assay//qRT-PCR//Western blot		O			
hsa-miR-376c-3p	BCL2	4	1	Flow//Immunohistochemistry//qRT-PCR//Western blot	O				
hsa-miR-376c-3p	ACVR1C	4	2	Luciferase reporter assay//Western blot//Reporter assay;Western blot;qRT-PCR//qRT-PCR				O	
hsa-miR-376c-3p	GRB2	3	1	Luciferase reporter assay//qRT-PCR//Western blot		O			
hsa-miR-376c-3p	TGFBR1	3	1	Luciferase reporter assay//qRT-PCR//Western blot				O	
hsa-miR-376c-3p	UGT2B15	2	2	Luciferase reporter assay//qRT-PCR					O

hsa-miR-376c-3p	UGT2B17	2	2	Luciferase reporter assay//qRT-PCR					O
hsa-miR-584-5p	ROCK1	5	1	Immunohistochemistry//Luciferase reporter assay//Microarray//qRT-PCR//Western blot				O	
hsa-miR-7-5p	BAX	1	1	Luciferase reporter assay	O				
hsa-miR-7-5p	BCL2	5	2	GFP reporter assay//Luciferase reporter assay//qRT-PCR//Western blot//Reporter assay;Western blot;qRT-PCR;Other	O				
hsa-miR-7-5p	CCNE1	4	2	Flow//Luciferase reporter assay//qRT-PCR//Western blot	O				
hsa-miR-7-5p	SERPINB5	7	2	Immunoprecipitaion//Luciferase reporter assay//Microarray//qRT-PCR//Western blot//ChIP-seq//Immunohistochemistry	O				
hsa-miR-7-5p	EGFR	11	12	real-time RT-PCR//Reporter assay//Western blot//Luciferase reporter assay//Reporter assay;Western blot;qRT-PCR;Other//qRT-PCR//Reporter assay;Western blot;qRT-PCR;Microarray;Other//Microarray//Immunohistochemistry//In situ hybridization//Flow		O			
hsa-miR-7-5p	FOS	8	1	ChIP-seq//Immunofluorescence//Immunohistochemistry//In situ hybridization//Luciferase reporter assay//Northern blot//qRT-PCR//Western blot			O		
hsa-miR-7-5p	GDF5	3	1	Luciferase reporter assay//qRT-PCR//Western blot				O	
hsa-miR-7-5p	PIK3CD	3	2	Luciferase reporter assay//qRT-PCR//Western blot		O			
hsa-miR-7-5p	PIK3R3	4	1	ELISA//Luciferase reporter assay//qRT-PCR//Western blot		O			
hsa-miR-7-5p	RAF1	5	2	Luciferase reporter assay//Microarray//qRT-PCR//Western blot//Reporter assay;qRT-PCR;Microarray;Other		O			
hsa-miR-7-5p	RELA	8	3	qRT-PCR//Western blot//ChIP-seq//Immunofluorescence//Immunohistochemistry//In situ hybridization//Luciferase reporter assay//Northern blot			O		
